# Blending Electronics with the Human Body: A Pathway toward a Cybernetic Future

**DOI:** 10.1002/advs.201700931

**Published:** 2018-08-01

**Authors:** Mehdi Mehrali, Sara Bagherifard, Mohsen Akbari, Ashish Thakur, Bahram Mirani, Mohammad Mehrali, Masoud Hasany, Gorka Orive, Paramita Das, Jenny Emneus, Thomas L. Andresen, Alireza Dolatshahi‐Pirouz

**Affiliations:** ^1^ Technical University of Denmark DTU Nanotech Center for Nanomedicine and Theranostics 2800 Kgs Denmark; ^2^ Department of Mechanical Engineering Politecnico di Milano 20156 Milan Italy; ^3^ Laboratory for Innovations in MicroEngineering (LiME) Department of Mechanical Engineering University of Victoria Victoria BC V8P 5C2 Canada; ^4^ Center for Biomedical Research University of Victoria Victoria V8P 5C2 Canada; ^5^ Center for Advanced Materials and Related Technologies (CAMTEC) University of Victoria Victoria V8P 5C2 Canada; ^6^ Process and Energy Department Delft University of Technology Leeghwaterstraat 39 2628 CB Delft The Netherlands; ^7^ NanoBioCel Group Laboratory of Pharmaceutics School of Pharmacy University of the Basque Country UPV/EHU Paseo de la Universidad 7 01006 Vitoria‐Gasteiz Spain; ^8^ Biomedical Research Networking Centre in Bioengineering, Biomaterials, and Nanomedicine (CIBER‐BBN) Vitoria‐Gasteiz 28029 Spain; ^9^ University Institute for Regenerative Medicine and Oral Implantology—UIRMI (UPV/EHU‐Fundación Eduardo Anitua) Vitoria 01007 Spain; ^10^ School of Chemical and Biomedical Engineering Nanyang Technological University 62 Nanyang Drive Singapore 637459 Singapore; ^11^ Technical University of Denmark DTU Nanotech 2800 Kgs Denmark

**Keywords:** conductive polymers, cyborganics, flexible bioelectronics, nanomaterials, wearable healthcare monitors

## Abstract

At the crossroads of chemistry, electronics, mechanical engineering, polymer science, biology, tissue engineering, computer science, and materials science, electrical devices are currently being engineered that blend directly within organs and tissues. These sophisticated devices are mediators, recorders, and stimulators of electricity with the capacity to monitor important electrophysiological events, replace disabled body parts, or even stimulate tissues to overcome their current limitations. They are therefore capable of leading humanity forward into the age of cyborgs, a time in which human biology can be hacked at will to yield beings with abilities beyond their natural capabilities. The resulting advances have been made possible by the emergence of conformal and soft electronic materials that can readily integrate with the curvilinear, dynamic, delicate, and flexible human body. This article discusses the recent rapid pace of development in the field of cybernetics with special emphasis on the important role that flexible and electrically active materials have played therein.

## 1 Introduction

**Figure 1:**
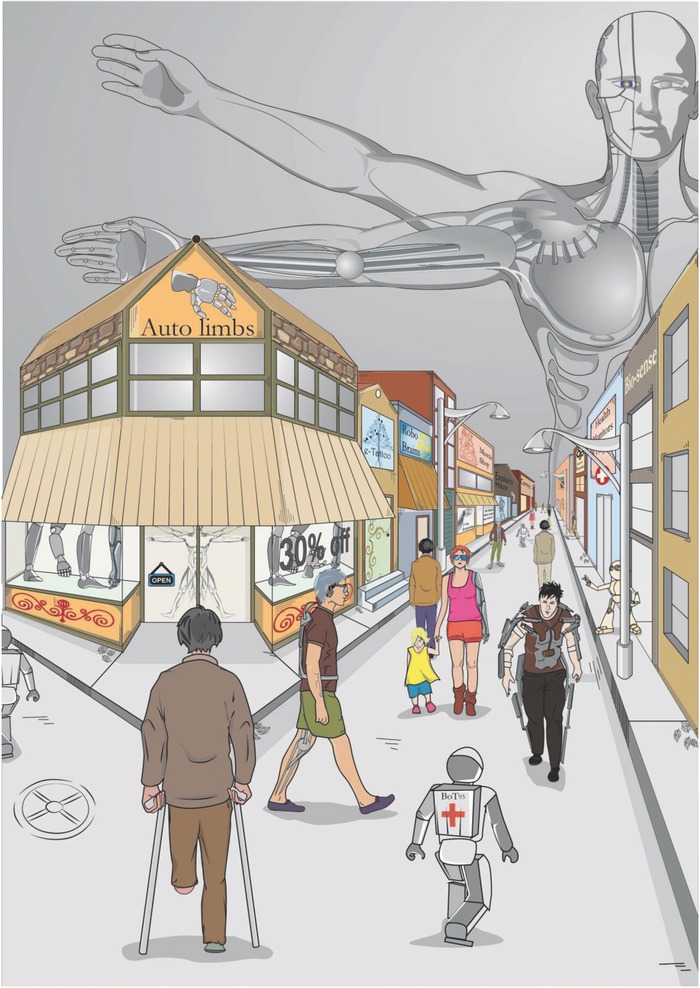
Recent innovations in materials science are leading humanity on the road to a cybernetic future—a future wherein, the fine‐line between machine and human will slowly fade away and pave the way for cyborg‐like humans.

The field of cybernetics emerged in the early 1960s to describe the possible merging of inanimate materials with living organisms.^1^ The original definition of cybernetics has subsequently been expanded to also encompass technical healthcare monitors for the health‐conscious consumer and implants for the sick and disabled. Indeed, throughout the course of history, the survival of mankind has relied on its ability to develop materials such as textiles, alloys, metals, and various types of rubbers, gums, and clays that could address our biological limitations in relation to a sustainable livelihood in the unfriendly and changing habitats of the Earth. In a broad sense, cybernetics therefore represents an expansion of the material‐making industries of the past. However, in striking contrast to ordinary materials, cybernetic extensions are items that can integrate with the body to overcome the limitations of human biology in an even more daring manner compared to external clothing, tools, or machines for that matter. Therefore, cyborgs (short for “cybernetic organisms”) could ultimately be the logical evolution of humans into a more adaptable, smarter, and stronger organism (**Figure** **1**).

**Figure 2:**
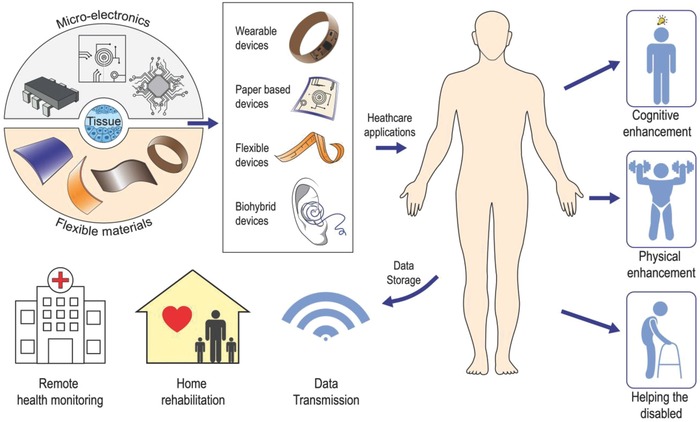
The union of microelectronics, flexible materials, and living tissues has led to a great variety of cybernetic devices that can bring relief to the lives of those disabled from disease or aging, enhance the physical capabilities of human beings beyond normality, and expand human consciousness toward uncharted territories.

These cybernetic extensions can monitor physiological signals, stimulate tissues, restore lost tissue functions, or even impose new superhuman abilities in their user. A great variety of cybernetic concepts are currently under development, some have already hit the healthcare market, while others are being carefully evaluated in laboratories all over the world.^2^, ^3^, ^4^ Classified broadly, these devices can be organized into three major categories: a) wearable healthcare monitors that can provide the user with individualized health information; b) prosthetics that can replace disabled organs or body parts; and c) implants that possess the ability to transcend human biology beyond its current limitations (**Figure** **2**).

The manufacturing of the abovementioned cybernetics relies on a number of challenges that have only recently begun to be addressed in depth. First, one of the key requirements of any device intended to interface with the human body is a proper integration between the device and the body to assure long‐term performance. To this end, a number of challenges related to the mechanical mismatch between conventional rigid electronic materials and the dynamic, soft, and curvilinear human body need to be solved as the elastic moduli of silicon and metal‐based electronics range from 1–170 GPa,^5^ which are in a stark contrast to the moduli of soft tissues in the order of 1–1000 kPa.^6^ Moreover, most of these electronic parts are made on 2D rigid silicon wafers with sharp edges that do not conform well to the curved human body. Finally, conventional rigid electronics are not flexible and typically break at ultralow strains (≈1%),^7^ and therefore, they are unable to withstand the high physiological strains of organs and tissues. The mismatch between conventional electronics and the human body ultimately hinders a good physiological contact with biological tissues and is thus a limiting factor for proper device performance. To remedy the current situation, flexible bioelectronics have emerged, which enable an unusually facile and intimate integration with biological tissues, such as skin, heart, and brain. Notably, they have been applied as brain–machine interfaces^8^, ^9^ to treat various neurological disorders, for real‐time monitoring of the beating heart,^10^, ^11^ as skin‐based devices that can monitor important health‐markers^12^, ^13^ and sophisticated prosthetics with the ability to reestablish a normal life for the hearing disabled,^14^ the paralyzed,^15^ and the blind.^16^ These devices are unequivocally opening new possibilities for studying chronic diseases, improving surgical procedures, and empowering patients to self‐manage their own health. Building on these groundbreaking results, bioengineers from Harvard University have recently begun to interweave living tissues within intricate nanowire‐based materials to create hybrid constructs that are half man, half machine,^17^, ^18^ or cyborg organic constructs (cyborganics) as the authors prefer to refer to them.

Overall, the gateway into the field of flexible bioelectronics is currently heavily related to the development of new multifunctional materials, which are flexible, resilient, and electrically active at the same time. In this review, we will highlight the recent advances of such electronic and flexible materials with a special focus on how these formidable materials currently are being used by scientist all over the world to reshape the field of cybernetics into a readily implementable technology. To this end, we will exclusively focus on healthcare monitoring devices, cybernetic prosthetics, brain–machine interfaces, and the exciting emergence of cyborganics. The review will begin with a brief overview of flexible and conductive polymers, since these materials are instrumental in the engineering of flexible bioelectronics. We will then proceed to discuss the incorporation of liquid metals and 2D nanoelectronic materials into such polymers, as the research and development of such composite systems in our opinion will soon lead to a number of scientific breakthroughs in the field of cybernetics. We will initially focus on the application of these materials in wearable and implantable healthcare monitors before highlighting their use in cybernetic prosthetics. As a new standpoint, we have also reviewed the recent progress in the development of cyborganics and the possible new directions that this emerging field might spearhead in the future. Finally, some of the current material‐based limitations in the field of cybernetics and how they can be successfully addressed will be discussed.

## 2 Flexible and Insulating Polymers

**Figure 3:**
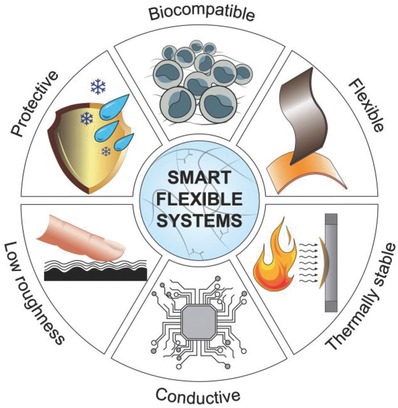
Special materials with special properties are required for proper interfacing between the electrical and biological components of cybernetic devices.

Flexible bioelectronics enable a great variety of biomedical applications that would otherwise be impossible to achieve using conventional rigid electronics, as these devices need to conform well to the human body in order to yield high‐quality recordings of physiological events.^19^, ^20^ They also need to sustain their operational capacity in the dynamic in vivo environment without succumbing to the cyclic movements of tissues and organs. For these reasons, materials used in flexible bioelectronics must have exceptional flexibility, toughness, and good biocompatibility to blend with the human body.^7^ Optical transparency is also instrumental in bioelectronics, as it makes wearable devices fit in visually with the human body, while at the same time providing the means for applications in which imaging is combined with multifunctional sensing. Polymers are promising material candidates in flexible bioelectronics, as they have many key advantages, including transparency, biocompatibility, and flexibility (**Figure** **3**).^21^, ^22^, ^23^ In addition, polymers are inexpensive, scalable via roll‐to‐roll processing, and enable reasonable tradeoffs in optical transparency, chemical performance, and thermal stability compared to metals and glass‐based materials.^24^ Flexible polymers are therefore currently considered as the material of choice for flexible bioelectronics, and thus they are the prime focus of this section.

### 2.1 Material Requirements

Electronic devices are mainly fabricated at high temperatures; this may lead to expansion or shrinkage due to internal stresses in the underlying polymeric material and therefore dimensional instability of the device.^24^ The relation between the thermal stability and device operation has made the coefficient of thermal expansion (CTE) and glass transition temperature important parameters for polymer materials in electronics. Obviously, the glass transition temperature of potential polymer candidates must be compatible with the process temperature to avoid dimensional changes during fabrication, while the CTE of the polymer should match with the low CTE of the conductive layers of the device to prevent strain accumulation and cracking during the fabrication process. Hence, polymers with a high glass transition point and low CTE are desired for flexible electronics.^24^

Another key requirement is a high barrier property because moisture and gas permeation may lead to dimensional instability of the polymer material. Still, there are many applications for flexible bioelectronics where the materials need not to be fully impermeable—but semi‐permeable—to allow biosensors to operate, such as in smart bandages.^25^ Polymers are also exposed to various chemicals and solvents during coating, patterning, etching, and other manufacturing processes and must have good chemical and solvent resistance.^26^

Surface roughness is another important parameter in the fabrication of flexible electronics, since the current transport in such devices is limited by surface defects and cracks.^26^ In a similar way, the surface defects can also catalyze crack formation when the device is bent and provide an easy pathway for unwanted diffusion of moisture and oxygen into the device.^24^ Overall, a number of different organic or inorganic barrier coatings such as paralyne C, polyethylene terephthalate (PET), aluminum and indium tin oxides have been used to prevent solvent or moisture diffusion into flexible electronics, reduce surface roughness, and improve the adhesion between the electronic parts and polymer.^26^

### 2.2 Amorphous Polymers

Polymers are essentially made from long chains of repeating monomer units held together by strong intermolecular interactions.^27^ The intermolecular interaction type, which varies a lot from polymer to polymer, plays a prominent role in many polymer properties. Depending on whether the polymer chains are nicely ordered or disorganized, polymers are also classified as either crystalline, semi‐crystalline, or amorphous.^28^ Notably, amorphous polymers typically exhibit a crystallinity <10% and crystalline polymers typically contain more than 80% crystalline structures, while semi‐crystalline polymers display a crystallinity ranging from 10% to 80%.^29^ They are also categorized into these different groups in terms of their glass transition temperature (*T*_g_) and melting temperature (*T*_m_). In this respect, the amorphous polymers have no clear melting point and their glass transition temperatures are approximately between −125 and 350 °C. On the other hand, semi‐crystalline polymers have a distinct *T*_m_, while their *T*_g_ in general is higher than amorphous polymers and typically in the range 75–260 °C.^24^ Highly crystalline polymers, however, only exhibit a *T*_m_ and do not have any *T*_g_ associated to them. Crystalline polymers can especially have extraordinary properties such as high stability, strength, and good resistance toward the surrounding environment.^30^, ^31^ These incredible properties of crystalline polymers are mainly associated with their well‐ordered molecular arrangements. For instance, such arrangements can severely restrict the molecular chain mobility, which in turn makes the polymer stronger mechanically and more rigid. However, in spite of this, crystalline polymers are in most cases not suited as materials for flexible electronics because of their rigidity, high surface roughness, and opaqueness.^24^, ^32^ To this end, the transparencies of polymers are intimately linked to their crystallinity degree, due to the intensified light scattering taking place within crystalline regions.^33^ Moreover, crystalline polymers have higher surface roughness due to the presence of growth facets on the crystallites. For these reasons, amorphous polymers are typically a preferred choice over crystalline polymers in flexible bioelectronics, as they are excellent transmitters of light, stretchable, and display smooth surface topographies. However, this comes at a high cost in terms of low barrier properties, dimensional instability, and high CTE coefficients.^24^ Examples of amorphous polymers include polycarbonate and polyethersulfone, which have high optical transparency and good flexibility but show poor solvent resistance, high gas permeability, and dimensional instability at high temperatures.^24^ In general, the CTE, optical transparency, and gas barrier properties of such amorphous polymers typically range between 54 and 75 ppm °C^−1^, 89–92%, and 50–80 g m^−2^ d^−1^, respectively.^24^ These listed values are relatively higher than that of semi‐crystalline polymers.^24^ Moreover, amorphous polymers exhibit modulus, flexural strength, and tensile strength in the range of 2.1–2.6 GPa, 93–115 MPa, and 50–84 MPa, respectively.^34^, ^35^, ^36^ They tend to bend and deform, but are not highly stretchable, as they typically only can stretch up to 60–200%.^36^

Polydimethylsiloxane (PDMS) is another interesting amorphous polymer that has found wide usage in bioelectronics due to its biocompatibility, nontoxicity, excellent optical transparency, and reasonable chemical resistance.^22^, ^37^, ^38^ Due to its very low glass transition temperature (*T*_g_ ≈ −125 °C) PDMS is a highly flexible and stretchable (≈1000%) material with a relatively low shear modulus ranging between 100 kPa and 3 MPa and flexural modulus ≈54 MPa.^39^, ^40^, ^41^, ^42^, ^43^ Importantly, the mechanical properties of PDMS are intimately linked with the way it is manufactured,^44^ and it displays an unusually high CTE value (≈310 ppm °C^−1^) as compared to other amorphous polymers.^45^ To date, PDMS has been used in many biological applications such as wound‐bandages,^46^, ^47^ microfluidics,^48^, ^49^, ^50^ and recently in various organs‐on‐a‐chip platforms.^51^, ^52^

Polyimides (PIs) are an interesting class of amorphous polymers that offer most of the advantages of semi‐crystalline polymers along with a high glass transition temperature (350 °C) and low CTE (8–20 ppm °C^−1^).^26^ They are also remarkably strong (2–4 GPa), flexible, and have an astonishing resistance toward chemicals and heat.^26^, ^53^ PI has been used in the electronics industry for decades and has begun to gain momentum as a polymer for flexible bioelectronics in recent years.^9^, ^13^, ^54^ Despite, the many advantages that PI‐based materials offer, they are not optically transparent (30–60%).^24^ However, current cutting‐edge technology has enabled the incorporation of fluorine, sulfonic, or nonaromatic groups into PI to make it optically transparent.^55^ PI‐based materials therefore hold great promise for flexible bioelectronics and have in recent years significantly advanced the field.

### 2.3 Semi‐Crystalline Polymers

Semi‐crystalline polymers are the most widely used group of polymers because they have unique properties that combine the best characteristics of amorphous and crystalline polymers.^56^ Semi‐crystalline polymers are typically very flexible, whereas highly crystalline polymers are rigid, as briefly mentioned in the previous section.^31^ Semi‐crystalline polymers also have a sharp melting point and do not soften gradually like amorphous polymers, which is a contributing factor to their superior dimensional stability at elevated temperatures as compared to amorphous polymers.^24^ However, the sudden phase transition above the glass transition temperature significantly impairs the temperature operating range of semi‐crystalline polymers.

Various semi‐crystalline polymers can be used for bioelectronics including PET, polyethylene naphthalate (PEN), and polyetheretherketone (PEEK).^24^ These semi‐crystalline polymers show desirable mechanical flexibility, solvent resistance, high clarity, low CTE (≈20–45 ppm °C^−1^), and good moisture barrier properties (0.1–0.5%).^24^ Notably, the mechanical and physical properties of semi‐crystalline polymers strongly depend on their morphology and the crystalline content within them.^57^ Their elastic modulus, flexural strength, and tensile strength have been reported in the range of 2.3–5 GPa, 70–170 MPa, and 55–200 MPa, respectively.^36^, ^58^, ^59^, ^60^ Importantly, they are highly flexible and bendable, and can stretch up to 50–300%.^36^ Although semi‐crystalline polymers have many of the aforementioned advantages, they do not possess the required upper operating temperature to match with the processing temperature of conventional electronic circuits (up to 350 °C),^24^ since PET, PEN, and PEEK have glass transition temperatures in the range of 80–150 °C.^26^ Another disadvantage of semi‐crystalline polymers is their high surface roughness, which significantly limits their performance in a range of applications.^24^ However, flexible bioelectronics such as transparent electrodes, sensors, and actuators based on PET^61^, ^62^, ^63^ and PEEK^64^, ^65^ materials with acceptable performances have still been successfully fabricated. Parylene C is yet another promising semi‐crystalline polymer for flexible bioelectronics, as it is biocompatible^66^ with a high *T*_g_ point (80–100 °C), low CTE (35 ppm °C^−1^),^24^, ^26^, ^67^ and displays excellent barrier properties toward water vapor, corrosive molecules, and various gases.^68^, ^69^, ^70^ Moreover, parylene C is a low‐cost polymer that can be easily processed into a protective ultrathin layer on almost any material, making it an ideal choice for the fabrication of cheap and ultrathin conformal bioelectronics.^71^, ^72^

Due to a growing technological demand, a significant amount of electronics are discarded and trashed every year.^73^ There are serious environmental concerns about the hazardous and toxic materials present in such discarded electronics.^74^ Therefore, the field is moving toward fabrication of flexible bioelectronics based on biodegradable and nontoxic materials in order to reduce the consequent accumulation of toxic waste. Flexible bioelectronics that can dissolve inside the body are also gaining momentum, as they can help to reduce the electronic waste materials and assure swift elimination of the bioelectronics once their mission in the body is accomplished.^75^, ^76^

Extensive research has been done on flexible and degradable electronics made from cellulose (paper) and silk‐based biopolymers. The degradation pathway of cellulose is typically driven through hydrolysis mediated by the family of cellulose enzymes, whereas silk—in its low‐crystalline state—is a highly water‐soluble protein, which disperses in physiological relevant solutions. Out of these materials, paper is particularly recognized for being a good material candidate in flexible electronics due to its high availability, flexibility, light weight, and sustainability.^77^ Various electronic items such as thin film transistors,^78^, ^79^ organic solar cells,^80^, ^81^ disposable radio frequency identification tags,^82^ batteries,^83^ and wearable diagnostic devices^2^, ^84^ have been made from paper‐based materials. Nanocellulose^85^, ^86^, ^87^ and silk^88^, ^89^, ^90^, ^91^ are also interesting biopolymers, as they are simultaneously strong, flexible, biocompatible, thermally stable, and recyclable at the same time. For instance, the decomposition temperature of nanocellulose has, in some instances, been shown to exceed 300 °C,^92^ while CTE values of around 8 ppm K^−1^ have been reported,^93^, ^94^ which are in the same range as that of glass^95^ and metals^96^ and are significantly lower than most commercial plastics (>200 ppm K^−1^).^26^ The amazing properties of nanocellulose have prompted its use as a material in electronics, with device performance comparable to its rigid counterparts.^97^, ^98^, ^99^ Silk also displays similar remarkable thermal and mechanical properties as nanocelluose, and in addition to its biodegradability, it can be easily tuned, making it a prime candidate in implantable bioelectronics.^10^, ^75^ Therefore, there is no doubt that various paper formats and biopolymers will open new avenues for fabrication of high‐performance flexible bioelectronics that are cost‐effective, nontoxic, biodegradable, and eco‐friendly.

## 3 Polymeric Conductors and Semiconductors

Many important biological processes such as neuronal activity,^100^ the synchronous beating of heart,^101^, ^102^ and muscle contraction^103^ are all tightly controlled by electricity. For this reason, most bioelectronic devices base their functionality on the conversion of biological signals into electricity^104^, ^105^—a signal format that can be easily recorded, modulated, and analyzed—and thus enable a great variety of bioelectronics. Electrodes play a major role in this direction, as they can connect the world of electronics with the world of living tissues. Among the many materials, gold (Au) and platinum (Pt) electrodes are still the standard choice in bioelectronics; however, their high cost and low flexibility represent critical issues for their application in flexible bioelectronics. Over the years, significant progress has been made by chemists to develop conductive, biocompatible, and flexible polymers that can address the aforementioned challenges. These multifunctional polymers are beyond doubt the primus motor behind the exponential speed at which the field of bioelectronics is currently evolving with. Therefore, the main focus of this section is directed toward conductive polymers.

### 3.1 Conductive Polymers

Electrically active polymers have emerged as a new class of mechanically robust and biocompatible materials for flexible bioelectronics.^20^, ^106^, ^107^ One of the most remarkable properties of these conductive polymers are the many functionalization pathways that they offer to fine‐tune the electrical and mechanical properties. They also offer other interesting features because some of them have intrinsic semiconductor properties that are essential for the design of basic electronic components such as transistors and field‐effect transistors.^108^ Due to their formidable multifunctional properties, the research and development of new polymeric conductors has dramatically expanded during the last decades; the most‐studied ones being polypyrrole (PPY), polyaniline (PANI), and poly(3,4‐ethylenedioxythiophene) (PEDOT).

#### 3.1.1 PPY

PPY is considered to be one of the pioneering polymeric conductors in bioelectronics due to its ease of functionalization and unique electrochemical properties (high conductivity and stability in oxidized states).^105^, ^109^ Depending on the conditions and reagents utilized in the oxidation, the electrical conductivity of PPy can range from 10 to 100 S cm^−1^.^110^, ^111^ It has been widely used as the outer electrode material for neural implants^112^ and for recording neural impulses.^105^, ^113^ PPY has also been used as a material for controlling cell‐fate through electrical stimulation and is recognized for its ability to yield high‐resolution electrical recordings from electroactive tissues and cells.^105^ Despite of their exciting electrical properties most PPy films are brittle and mechanically unstable due to their conjugated chain structure, which significantly limits their usage in many flexible electronics applications.^114^ To remedy this drawback, efforts have been dedicated for enhancing the strength of the PPy matrix by optimizing its manufacturing process^115^, ^116^, ^117^, ^118^ and reinforcing it with other polymers.^114^, ^119^, ^120^

#### 3.1.2 PANI

PANI was discovered as early as 1862,^121^ but it was not before the beginning of the 1980s that it started to garner the attention of scientists in the medical sciences.^122^, ^123^ Due to its exceptional stability,^124^ reasonable biocompatibility,^125^, ^126^, ^127^ and high conductivity,^128^ its range of applications encompasses a variety of fields including biomedical engineering,^129^ flexible electronics,^130^, ^131^ and electromechanical engineering.^132^ Moreover, because of PANI's good biocompatibility and high conductivity, it also holds great promise for use in the engineering of electroactive tissues.^133^, ^134^ To this end, the electrical conductivity of PANI has been reported within the range of 10–100 S cm^−1^; a parameter that can be fine‐tuned through molecular weight,^135^ temperature,^136^oxidation level,^137^ crystallinity degree,^138^ degree of doping,^139^ and film morphology.^137^, ^140^ However, unfortunately, since PANI is synthesized from acidic solutions, it tends to degrade in physiological environments significantly impacting its electrical stability in such environments.^141^ One possibility to address this problem is to functionalize PANI with specific dopants via either noncovalent or covalent approaches, or by utilizing PANI nanomaterials such as nanowires, nanofibers, and nanorods instead.^142^

#### 3.1.3 PEDOT

PEDOT is perhaps the most‐investigated electroactive polymer to date,^129^, ^143^ as it keeps its ability to conduct electricity over a broad pH range.^144^ It is also electronically stable in physiological environments^105^, ^145^ and can merge with in vivo tissues without inducing toxic and foreign body responses.^46^, ^146^ The polymeric backbone of PEDOT can also be easily functionalized to increase its conductivity, biocompatibility, and stability through the incorporation of various dopants, counter ions, and biological moieties.^147^, ^148^ The most frequently used PEDOT derivative is PEDOT doped with poly(styrene‐sulfonate) (PSS)—PEDOT:PSS—an optically transparent polymer with an electrical conductivity that can go as high as 4600 S cm^−1^.^149^ Even though, PEDOT:PSS films can be stretched up to ≈60%, the electrical conductivity in such strain regimes is highly compromised, and ultimately can present a great hindrance for the utilization of PEDOT:PSS in flexible electronics.^150^ Recently, this grand challenge has been tackled by incorporating ionic additives and various electrical conductivity enhancers to generate highly conductive and stretchable PEDOT:PSS films.^151^ These materials display a conductivity that can reach 4100 S cm^−1^ under 100% strains, because of their enhanced crystallinity and more interconnected polymeric networks.^151^

#### 3.1.4 Poly(3‐hexylthiophene) (P3HT)

P3HT is another interesting semi‐conducting polymer, which has been widely used in various types of electronics—in particular organic solar cells—since its discovery in 1980.^152^, ^153^ P3HT is easy to modify, nontoxic, and conductive, and is typically generated from monomers of 2,5‐polythiopene (2,5‐PT) by using various metals to initiate the polymerization process.^153^ Besides its usage in solar cells, P3HT has also recently found its ways into the field of flexible bioelectronics, as it has been used in several flexible electronic devices capable of sensing various biomolecules.^154^

### 3.2 Semiconductors

Replacing inorganic semiconductors with their organic counterparts involves a number of trade‐offs. The advantages include decreased manufacturing expenses, higher flexibility, and light‐weight.^108^, ^155^, ^156^ On the negative side, however, organic semiconductors can be electrically unstable in physiological environments,^157^, ^158^ they are more fragile than their inorganic counterparts,^108^, ^159^, ^160^ and they display a weak long‐term in vivo performance due to faster biodegradation in the body.

Over the years, several modification strategies aimed at strengthening intermolecular polymer bonds have been used to increase the stability of organic semiconductors. Especially, PEDOT:PSS has garnered significant attention, as it can be easily functionalized and is susceptible to electrical dopants.^147^ PEDOT:PSS is a p‐type organic semiconductor that is very sensitive to the surrounding electrolyte concentrations,^156^ and it is able to electrically respond to electrolytes through its amazing cation uptake ability.^161^ This property provides PEDOT:PSS devices with an unusually facile pathway to measure the many electrolyte‐sensitive events inside physiological environments. The unique electrical properties of PEDOT‐based polymers have sparked tremendous interest in the past few years with the primary focus being directed toward their applications as organic bipolar junction transistors and field‐effect transistors.^105^, ^156^

**Figure 4:**
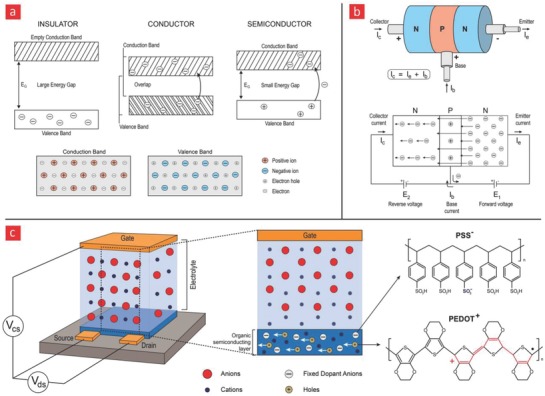
Energy bands and semiconductor‐related devices. a) Energy‐band diagrams for insulators, conductors, and semiconductors. The current in semiconductors are either generated from electrons (−) or electron holes (+). b) A transistor is basically made from three different semiconductors. The current can only run in one direction in a transistor, and the current that passes through it is typically enhanced with a sustainable gain factor, making them suitable for various sensing applications. c) A qualitative illustration of the working principles behind a sensor that is based on a field‐effect transistor made from PEDOT:PSS, which is an organic semiconductor capable of absorbing electrolytes (anions) from a solution. The uptake of anions abolishes the mobile holes within PEDOT:PSS and thus changes the current that passes through it; it thereby enables it to sense biological processes that either diminish or increase the amount of electrolytes in the surrounding environment.

In simple terms, a bipolar junction transistor consists of three semiconductors: a collector, base, and an emitter.^162^ A field‐effect transistor (FET) is a further extension of a transistor, as its operating principle is basically the same as a transistor with one exception, namely, the inclusion of an electrode (gate) above the transistor channel (base).^162^ By regulating the gate voltage, it is possible to control the current through the transistor channel and into the source. FETs therefore possess an extra feature compared to an ordinary transistor because of their ability to switch between two states, that is, a low current state (off) and a high current state (on) (**Figure** **4**). In most bioelectronic applications, the channel and the gate are separated by an electrolyte solution. By applying a positive potential to the gate, the “on” and “off” states, and ultimately the current across the channel, can be tightly regulated, as the positive gate potential drives cations from the electrolyte solution into the transistor channel (Figure 4), which in turn makes the FETs sensitive toward various forms of currents with a typical response time around 100 Hz. Another important electrical effect in some polymer‐based FETs is the formation of an electrical double layer, which can significantly improve the response time, as this double layer provides the FETs with high‐capacitance, and thus enables them to operate with much higher frequencies (≈10 kHz).^163^ This current sensitivity enables various sensing and detection schemes depending on the application. For instance, in a landmark study led by Malliaras and co‐workers, a simple but yet elegant PEDOT:PSS transistor was developed for glucose monitoring.^164^ The PEDOT:PSS transistor was capable of monitoring glucose concentrations through a mechanism that involved the enzymatic conversion of glucose into the byproducts gluconic acid and hydrogen peroxide (H_2_O_2_) by glucose oxidase. The formation of hydrogen peroxide was quite essential, as it significantly alters the gate potential resulting in a huge current drop across the transistor channel.^165^ In another study, the same principle was used to measure the lactate concentration in blood, which is a well‐known marker for monitoring the effect of exercise, wellness, and physical fitness.^166^ Instead of glucose oxidase, another enzyme (i.e., lactate oxidase) was used to convert lactate into pyruvate. The generation of pyruvate changed the gate voltage and thus the current across the transistor channel. Despite the many interesting applications of PEDOT:PSS‐based transistors, they are still behind inorganic transistors in terms of their conductivity and response time.

In summary, organic semiconductors hold great promise in flexible bioelectronics due to their reasonable sensitivity, high flexibility, biodegradability, and low cost. They have already successfully been used in a wide range of biological and medical applications, and further applications are expected once their response time and conductivity are made to match their inorganic counterparts.

## 4 Conductive Polymer Composites

One avenue to bridge the current gap between conductive polymers and their inorganic counterparts is to reinforce them with inorganic fillers. The prime components of most of today's inorganically reinforced polymers are 2D and 1D nanomaterials.^167^ A wide selection of such materials exists; the most studied ones are 2D graphene sheets and 1D carbon nanotubes (CNT) and silicon nanowires, however, other emerging nanomaterials are also worthy of consideration, such as boron nitride, silicene, germanene, and phosphorene.^167^ Owing to their remarkable electronic, thermal, piezoelectric, mechanical, and moisture‐sensing properties, these nanomaterials have found widespread importance in flexible electronics.^167^, ^168^, ^169^ Indeed, it is anticipated that their inclusion in electronics could revolutionize the entire industry and facilitate the emergence of better, faster, and smarter electronics that can be readily implemented within existing electronic formats.^168^ The unique portfolio of properties that nanomaterials bring to the table can also be readily utilized in flexible bioelectronics to yield even more flexible and electrosensitive devices.^169^, ^170^, ^171^

Another area that avenues to improve the electrical properties of flexible polymers is the incorporation of liquid metals into them, as liquid metals are conductive, self‐healing, and reconfigurable. It has therefore been foreseen that their incorporation into polymers can lead to sophisticated electronic circuits that can spontaneously repair upon damage.

Even though the research and development of the abovementioned inorganic fillers is still in its infancy, the achievements obtained to date are truly remarkable and have already surpassed those reported by conventional organic‐based polymers. We are therefore confident that these fillers will be a game‐changer in flexible bioelectronics. One concern, however, is their cytotoxicity and biocompatibility in vivo, which still has not been carefully evaluated. This is beyond doubt one of the major challenges that needs to be addressed before their potential can be translated into devices that can integrate seamlessly with the human body.

### 4.1 Graphene

In brief, graphene consists of a monolayer of carbon atoms that are packed densely into a 2D hexagonal honeycomb lattice with a carbon–carbon bond length of 1.42 Å^172^ and a thickness of only one atomic layer (≈0.3 nm), which makes it the “thinnest” material ever discovered.^173^ This ultrathin 2D nanomaterial displays really high electrical conductivity (2.50 × 10^5^ cm^2^ V^−1^ s^−1^),^174^ good thermal conductivity (≈3000 W m^−1^ K^−1^),^174^ amazing mechanical flexibility (ultimate tensile strength (130 GPa), high Young's modulus (≈1 TPa),^174^, ^175^ and low coefficient of thermal expansion (CTE) (≈−8 × 10^−6^ K^−1^).^176^, ^177^ Moreover, due to its high specific surface area (up to 2600 m^2^ g^−1^),^178^ large aspect ratio (up to 2000),^179^ chemical reactivity, and tunable interface properties, graphene is much easier to functionalize compared to many other nanomaterials.

**Figure 5:**
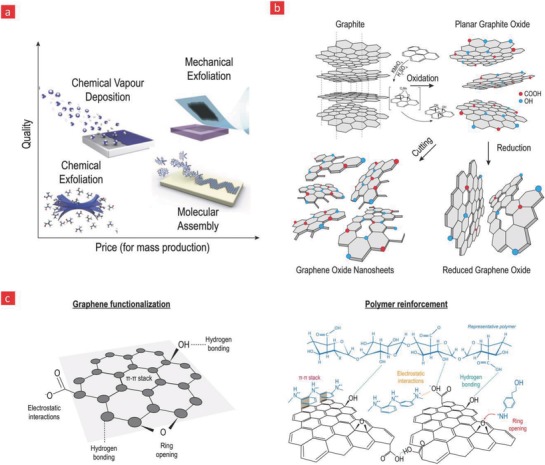
Various a) manufacturing methods of graphene. Reproduced with permission.^447^ Copyright 2012, Nature Publishing Group, b) graphene oxide, reduce graphene oxide, and graphene oxide nanosheets are highlighted here. c) Graphene contains numerous functionalities, which can be used to firmly attach it to the backbone of polymers.

Typically, graphene is synthesized by either top‐down or bottom‐up strategies.^180^ In the top‐down approach, graphene sheets are exfoliated from the bulk graphite via chemical reactions or mechanical forces to get single or few‐layers of graphene (**Figure** **5**). In the bottom‐up strategy, graphene nanosheets are directly grown onto a substrate—starting from single atoms or molecules—by using chemical vapor deposition (CVD), organic synthesis, or solvothermal synthesis methods.^181^, ^182^ Until now, different types of graphene have been successfully achieved by using these two strategies. These different varieties include graphene oxide (GO), graphene oxide quantum dots, graphene quantum dots, and reduced graphene oxide (rGO) (Figure 5).^182^, ^183^ Among them, GO has received most attention owing to its highly oxidized nature due to its large numbers of surface residual epoxides, hydroxyl, and carboxylic acid group, which in turn provides many chemically reactive groups for various functionalization purposes. GO can also easily be reduced into highly conductive graphene (rGO) by eliminating oxygen‐containing functional groups through chemical, thermal, or irradiation treatment.^183^

Given these unique features of graphene, it has attracted tremendous attention as an electroactive and mechanical nanoreinforcer with the capacity to turn nonconductive polymer‐based materials into amazing conductors of electricity with impressive mechanical properties.^184^, ^185^ However, it should be emphasized that the electrical conductivity and sensitivity of graphene are typically greatly affected by other factors such as the presence of adverse functional groups on the graphene sheets, various intrasheet and intersheet structures, intersheet junctions, the aspect ratio of the sheets, and the processing methods used to manufacture them.^184^, ^186^, ^187^ Overall, they are therefore potential candidates for the development of even better conductive and polymer‐based electrode–tissue interfaces for flexible bioelectronics.

As an example, graphene incorporation within PEDOT has resulted in significant improvement of mechanical and electrical properties, features that were used to enhance the performance of neural and microelectrode interfaces.^188^ In this case, the positive charge of oxidized PEDOT chains was ionically bonded to the negatively charged group of GO to form a stable conductive polymer film. An advantage of this functionalization strategy is that it prevents GO from dispersing into the target tissue during electro‐physiological recordings, and can thus minimize any possible cytotoxicity caused by graphene inside the body. Aside from chemical incorporation of graphene into PEDOT, graphene can also be physically mixed with PEDOT polymer chains to form a free‐standing film. This approach relies on the polymeric structure of PEDOT, which consists of many conjugated π bonds, that enable strong π–π stackings between PEDOT molecules and graphene sheets. For instance in one study, it was shown that such free‐standing graphene–PEDOT composite films can lead to an almost sixfold increase in mechanical strength and more than twofold improvement in electrical conductivity as compared to a pristine PEDOT polymer film.^189^

Graphene may also be used to effectively reinforce PEDOT:PSS polymer film for flexible bioelectronics. Toward this endeavor, studies have shown that the graphene–PEDOT:PSS composites can be formed by either in situ polymerization or various blending processes.^190^, ^191^ In the in situ polymerization method, graphene is typically dispersed in the PSS solution after which the EDOT monomer is gently added. Then, the polymerization process is initiated in the presence of a Fe^3+^.^190^ Compared to PEDOT:PSS film, the electrical conductivity of the graphene–PEDOT:PSS composite film was increased with up to 41% (637 S cm^−1^) at only 3 wt% graphene loading. Although this approach is effective for homogeneous dispersion of graphene sheets in the polymer matrix, it is limited, as the rate of polymerization is decreased at high graphene content. By contrast, the solution blending method is the most straightforward approach to use for developing polymer films at high graphene concentrations. To this end, Seol et al. recently developed a stretchable and transparent conducting electrode based on mixing PEDOT:PSS with rGO.^191^ Specifically, the authors managed to reduce the adverse agglomeration of rGO by functionalizing rGO with a surfactant—(phenyl isocyanate)—that could reduce possible π–π interactions between rGO nanosheets and PEDOT:PSS. This flexible composite system displayed significantly higher optical transmittance (≈86%) and a greater electrical conductivity (1010 S cm^−1^), as compared to rGO–PEDOT:PSS and pristine PEDOT:PSS.

Graphene has also in recent years been used in wearable sensor systems due to its ability to improve the accuracy in position, acceleration, and velocity detection of its wearer at high strains and strain rates.^192^, ^193^, ^194^ In an enlightening study, Boland et al. showed that by loading graphene into a natural rubber, it is possible to produce conducting composites with electrical conductivity as high as 0.1 S m^−1^.^193^ In this system, the graphene nanosheets could quickly respond to polymeric deformations caused by dynamic movements in a time dependent manner, due to mechanically induced changes in electrical conductivity within the polymeric matrix. Moreover, this flexible composite system could stretch up to 8 times its original length without losing electrical functionality and mechanical integrity. Other noteworthy application of graphene is its inclusion into piezoresistive/piezoelectric polymer‐based sensors for converting the kinetic energy of the moving body into harvestable energy and in electronic skin (e‐skin) devices.^169^, ^170^, ^171^

Other properties of graphene such as electrical and mechanical stability can be further coupled with control over the specific interactions between graphene and polymers to generate a self‐healing and conductive composite system.^195^ The impartation of self‐healing function empowers the electronics to be revamped not only mechanically but also electrically, which is of great interest for electrical functional restoration after damage during large mechanical strain regimes. To this end, graphene has been incorporated into a self‐healing polyvinyl alcohol (PVA) polymer matrix to generate highly stretchable and self‐healing strain sensors.^196^ The conductive graphene‐hydrogel based strain sensors displayed fast electrical healing speed (within 3.2 s), remarkable self‐healing performance (≈98%), and was able to sustain high elastic deformation (≈1000%) with gauge factor of 0.92.^196^ Although this system is unequivocally opening new possibilities for usage in robotics, healthcare monitoring, and various human motion detection systems, further efforts should be focused on enhancing their stability and sensitivity to bring this interesting technology into the mainstream market.

### 4.2 Carbon Nanotubes (CNTs)

**Figure 6:**
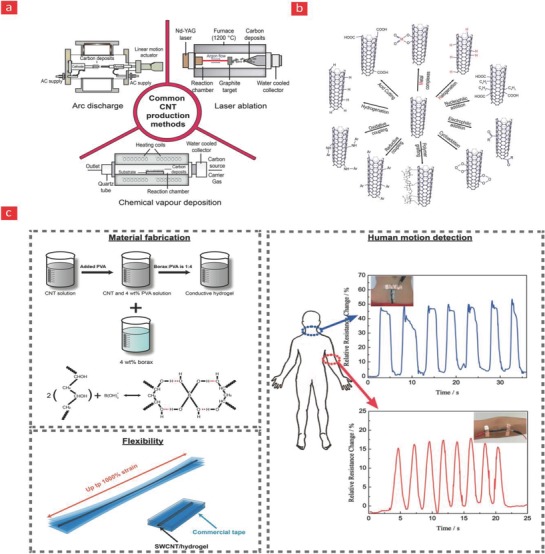
Various CNT a) production and b) functionalization strategies. c) A self‐healing and flexible PVA‐CNT based composite for human motion detection. Adapted with permission.^196^ Copyright 2017, Wiley‐VCH.

CNTs belong to the fullerene family of nanomaterial's and consist of graphene sheets that are rolled‐up into high‐aspect ratio tubes (>1000).^197^ They come both as single‐wall carbon nanotubes (SWCNTs) and multiwall carbon nanotubes (MWCNTs), and for the most part “the two” show similar properties, however, the tensile strength of SWCNTs is significantly lower than MWCNTs, which makes SWCNTs more flexible than their multiwalled counterpart.^198^ Over the years CNTs, have found widespread application in diverse fields ranging from electronics, medicine, and drug‐delivery, as they pose unique properties such as formidable tensile strength (11–63 GPa),^199^ high Young's modulus (1–1.8 TPa),^200^ excellent intrinsic conductivity (10^9^ A cm^−2^),^201^ high thermal conductivity (2000–6000 W m^−1^ K^−1^ at room temperature),^202^ and are thermally stable up to 2800 °C in vacuum conditions.^203^ They are also perfect reinforcers for flexible electronics, as they are readily bendable and squeezable, and display spring‐like properties under constant loading. Furthermore, CNTs can be synthesized via various methods with the most frequently applied ones being electrical arc‐discharge, laser ablation, and CVD (**Figure** **6**).^204^, ^205^, ^206^ With each of these methods, the surface chemistry, surface area, surface charge, and CNT size distribution can be uniquely fine‐tuned to yield desired CNT batches for further downstream applications.^204^ In laser ablation, laser pulses are applied to pure graphite blocks to vaporize them into ultrathin pieces that subsequently can generate CNTs on a water cooled collector,^204^ while in the electrical discharge process a high current is applied between two graphene electrodes—anode and cathode—in the presence of metallic catalysts, which catalyze the growth of CNTs on the cathode (Figure 6).^204^ The most standard method employed however is CVD, which is based on the pyrolysis of hydrocarbons in a tube furnace and the usage of metallic‐catalysts to polymerize them into CNTs. As the CVD method is the most practical, economical, and pure pathway for CNT synthesis, it is also the one most commonly used for commercial‐scale production of CNTs.^205^

The outstanding properties of CNTs have turned them into widely used nanofillers in polymer matrixes for the construction of flexible, stretchable, and deformable electronics.^22^, ^207^, ^208^, ^209^, ^210^ Until now, several methods have been proposed to incorporate CNTs into polymers such as solution mixing, melt processing, and in situ polymerization.^211^ However, untreated CNTs are chemically inert and too hydrophobic to disperse with ease in organic or inorganic solvents, and are therefore unable to establish strong interactions with the polymer backbone. The key to overcome this obstacle is the functionalization (covalent or noncovalent) of CNTs with hydrophilic groups such as hydroxyl, carbonyl, carboxyl, and amines to improve the dispersion stability and chemical reactivity of CNTs, and ultimately yield reinforced polymers with good electronic, magnetic, optical, thermal, and mechanical properties.^212^ Moreover, the performance of stretchable CNT‐based electronics could be enhanced by alignment of the nanomaterials. Indeed, studies have shown that horizontally or vertically aligned CNTs within polymer matrixes can significantly improve the conductivity and mechanical performance of flexible electronics.^213^, ^214^, ^215^, ^216^, ^217^

Notably, CNTs typically form a percolation network within the matrix, which in turn significantly enhances the electrical conductivity within the polymer. For instance, several studies have shown that CNTs networks can be formed within polymers to yield highly conductive and stretchable strain sensors.^22^, ^207^, ^208^, ^216^, ^218^, ^219^ In one paramount study, SWCNTs were embedded into a stacked nanohybrid structure within polyurethane (PU)–PEDOT:PSS to provide a transparent, stretchable, and patchable strain sensor.^208^ This strain sensor displayed good optical transparency (≈63%), a high gauge factor (62.3), and could stretch up to 100% before breakage. In a similar vein, SWCNTs were embedded into PDMS to generate a sensor that could sense mechanical deformations arising at the “bone–skin” interface to enable the detection of joint‐movements in the human body with a mechanical sensitivity ranging as low as 10^5^ MPa^−1^ at 0.9 MPa pressure.^22^ Despite of its incredible sensitivity this system, however, could only perform at strains at 30% as the conductive CNT percolation network otherwise would breakdown.

One avenue to improve the CNT percolation network is based on generating a PDMS foam consisting of 3D‐interconnected networks and then dip the entire polymeric foam into a CNT solution.^209^, ^220^ In this procedure the CNTs will adhere to the PDMS network to generate a percolated network of conductive wires. For example, in a recent groundbreaking study, this concept was successfully used to develop a highly sophisticated wearable strain sensor with an excellent gauge factor of 134 at a 40% strain value.^209^ Another strategy to achieve a more favorable CNT network within a polymer matrix is the use of graphene sheets to form a hybrid CNT/graphene structure, which efficiently inhibits the bending and bundling deformation of CNT networks during successive stretchings.^220^ In this scenario, the graphene nanosheets are able to disperse and adsorb pristine CNTs, whereas the CNTs are capable to function as bridges and avert the restacking of graphene nanosheets through π–π interactions.

As previously mentioned, the integration of self‐healing properties into polymers has speared a major paradigm shift toward the development of flexible and stretchable electronic devices with higher durability during successive loadings. Toward this endeavor, a self‐healing piezoresistive strain sensor device, which is able to detect the dynamic movement of the human body was recently developed by incorporating SWCNTs into a self‐healing PVA polymer matrix (Figure 6).^196^ The self‐healing mechanism was built into the system via reversible hydrogen bonds in the PVA matrix mediated by borate ions through a simple one‐pot mixing of CNT, PVA, and borax. These weak hydrogen bonds could easily break and reform, and were thus one of the main driving mechanisms behind the self‐healing properties of the manufactured device. Specifically, the device was incorporated into a Scotch permanent clear mounting tape, which acted as an elastomeric substrate, and enabled the system as a whole to stretch 1000% with an amazing self‐healing efficiency of 98%, after only 3 s of healing time. In addition to this formidable stretching ability, which according to the authors was the highest value ever recorded for such device, the SWCNTs themselves also established spring‐like and conductive links between the individual polymer chains, and thus contributed to a significant improvement of the elasticity, conductivity, and flexibility of the composite system relative to pristine PVA. The sensor device was then mounted onto human joints, and the authors demonstrated its capacity to monitor various human motions in real time through changes in resistivity brought about by the mechanical strains, that the PVA‐CNT polymer experienced during human joint motions.

### 4.3 Metallic Nanowires

Existing bioelectronic devices primarily use metal electrodes such as Au, Pt, and Ag because of their suitable electrical properties, biocompatibility and corrosion resistance.^221^, ^222^, ^223^, ^224^, ^225^, ^226^ Especially, Au has been extensively used for bioelectronics due to its high ductility, good corrosion resistance, good biocompatibility, and long‐term operational stability. Au‐based electrodes have found many interesting applications in bioelectronics; some of the most noteworthy are as electrical conductors for glucose biosensors,^227^ cochlear implants,^228^ and in electrodes that enable communication between the brain and various machine formats.^72^

For instance, Au nanostructures in the form of nanowires have gained great interests in flexible and stretchable bioelectronics, owing to their remarkable aspect ratio (≈10 000), mechanical and electrical properties.^229^, ^230^ To this end, Gong et al. fabricated a wearable and highly sensitive pressure sensor by sandwiching ultrathin gold nanowires (AuNWs) between PDMS sheets.^229^ This system was able to detect pressures as low as 13 Pa with a response time of <17 ms, and sensitivity of 1.14 kPa^−1^ in the pressure range of 0–5 kPa. Due to its excellent sensing, flexibility, and robustness, this device was used for real‐time monitoring of blood pressure and various acoustic vibrations. Although, AuNWs‐based devices are quite promising for the field of flexible electronics their conductivity could be improved even more. A possible approach for improving the conductivity of such devices is by combining them with conductive polymers. For example, PANI microparticles have been doped into AuNWs films to yield a tenfold improvement in conductivity and eightfold enhancement in electrical sensitivity in comparison to pristine AuNW‐based strain sensors.^231^

Silver nanowires (AgNWs) are another interesting class of metallic nanowires, which has been widely used in various types of wearable and flexible bioelectronics.^226^, ^232^, ^233^, ^234^ AgNWs can form highly conductive percolative networks to yield a good optical transparency and a high structural flexibility. For instance, Ho et al. developed a transparent stretchable strain sensor based on percolating networks of both AuNWs and AgNWs on an elastomeric PDMS substrate.^232^ This strain sensor displayed good optical transparency (≈66.7%), high gauge factor (≈236), and could stretch up to 70% of its original length. Due to their high conductivity, compatibility, and mechanical deformability, AgNWs‐based polymer‐based electronics have also been recognized as promising nanomaterials for electrophysiological recordings.^226^, ^235^ For instance, in a recent study, AgNWs were patterned into styrene–butadiene–styrene (SBS) elastomers to form serpentine‐like meshes capable of mimicking the elastic and electrical properties of cardiac tissue.^226^ In this scenario, the AgNWs formed a highly conductive percolation network, while the SBS rubber acted as a binder to maintain the mechanical elasticity. This stretchable cardiac mesh was readily integrated with the curvilinear and dynamic in vivo heart and could ultimately improve cardiac contractile function in a post‐myocardial‐infarction model.

Although, AgNWs and AuNWs have demonstrated promising results in the field of flexible electronics, they are relatively expensive compared to their organic counterparts Hence, copper nanowires (CuNWs) have become an appealing alternative, since they are cheaper, but yet, display an electrical conductivity similar to that of silver (Ag) and Au.^236^, ^237^ So far, different types of wearable and flexible devices have been developed by incorporating CuNWs into various elastic polymers, such as poly (acrylate),^238^ polyurethane,^239^ PVA,^240^ Eco‐flex,^241^ and SBS.^242^ Although, CuNWs have many of the aforementioned advantages, CuNWs display some disadvantages related to their easiness to become oxidized, which significantly limits their electrical performance in a range of applications as oxidative layers are highly insulating.^237^, ^239^ One strategy to overcome this obstacle is by coating CuNWs with corrosion‐resistant metals such as nickel, Pt or Ag.^237^, ^243^, ^244^ For example, Song et al. coated CuNWs with nickel (Ni) to improve their oxidation‐resistant stability.^237^ Subsequently, the CuNW–Ni composite was embedded into PDMS to provide a conductive elastomer composite with transparency of 80% and resistance of 62.4 ohm sq^−1^. This composite system could endure up to 600 cycles of bending, stretching, and twisting tests without breaking.

### 4.4 Silicon Nanowires

1D silicon nanowires (SiNWs) are also steadily gaining a foothold in bioelectronics—albeit to a lesser degree compared to graphene and CNTs—due to their unique electrical, mechanical, and optical properties.^245^, ^246^, ^247^ Indeed, compared to the other nanomaterials discussed here, SiNWs have an advantage in terms of their better semiconductor properties,^248^, ^249^ which in turn makes them amenable in the development of nanoscaled transistors and FETs.^250^, ^251^ Notably, the nanoscale diameter and high‐aspect ratio of silicon nanowires significantly alter, and in some cases improve their electrical properties as compared to solid silicon, due to quantum effects arising from quantum confinement within the wires. The small size of silicon nanowires also makes it much easier to control their electrical properties. For instance, one can significantly widen the band‐gap of silicon nanowires by simply decreasing their diameter and the orientation of the wire axis also have an important say on the many interesting properties of silicon nanowires.^252^, ^253^, ^254^

Various techniques based on both top‐down and bottom‐up manufacturing have over the years been developed to generate silicon nanowires.^245^, ^246^ In the bottom‐up process individual Si atoms are lined up into silicon nanowires with diameters between a few nanometers to several hundred nanometers via CVD and vapor–liquid–solid based methods. In the top‐down approach different lithography methods such as electron beam lithography and reactive‐ion etching are employed to carve out ultrathin silicon nanowires from solid silicon wafers. Even though, top‐down approaches are the most attractive to employ due to the high precision and flexibility they offer, these benefits come at a huge cost, as conventional lithography methods are time‐consuming, costly, and difficult to upscale to meet an industrial scale production.

Due to their amazing electronic properties, silicon nanowire devices display impressive sensitivity when it comes down to measuring bioelectrical signals in the body.^255^, ^256^ For instance, silicon nanowires have recently been employed in implantable bioelectronics with the purpose of enabling even better electrophysiological recordings as well as controlled drug release in response to important biological events inside the body.^15^, ^17^, ^257^ Despite of these interesting advances, which will be elaborated in a more detailed manner in Section 6.4, silicon nanowires have not been as widely used in the field of bioelectronics as compared to their carbon‐based counterparts, and the focus have for their part so far mainly been directed toward photovoltatic, electronic, and energy storage devices. The authors therefore anticipate that the incorporation of silicon nanowires into biocompatible polymers represent an interesting area ripe for investigations, and could therefore enable significant breakthroughs in the field.

### 4.5 Liquid Metals

**Figure 7:**
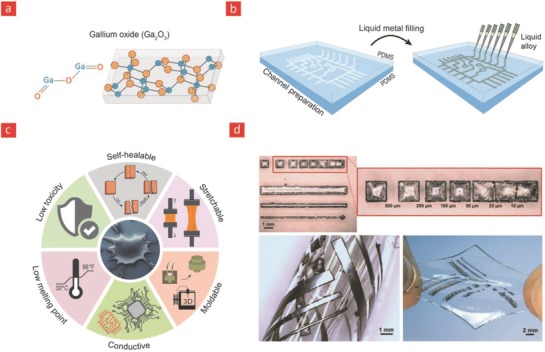
Gallium oxide, its properties, and application in flexible bioelectronics. a) The chemical structure of gallium oxide. b) The many unique properties that gallium oxide has to offer. c) Incorporation into polymers to yield flexible and electrical circuits. d) A gallium embedded PDMS substrate with high‐fidelity and stretchable circuits. Reproduced with permission.^272^ Copyright 2013, Wiley‐VCH.

Another class of inorganic materials for flexible electronics is liquid metals, as these materials are liquid near room temperature and therefore flow readily in response to stress (**Figure** **7**).^258^ This enables them to deform and stretch in response to stress in a reversible manner, which makes them ideal candidates for stretchable and self‐healable electronics.^259^, ^260^, ^261^ Especially, in recent years, there have been tremendous advancements in the emerging technology of stretchable electronics based on liquid metals such as mercury and gallium oxide (Ga_2_O_3_).^262^ However, mercury is a well‐known environmental toxicant,^263^ gallium oxide on the other hand has a relatively low toxicity making it ideal for flexible bioelectronics.^262^ Moreover, gallium has a number of interesting properties that makes gallium an attractive component to include in flexible bioelectronics,^264^, ^265^ as gallium has high electrical conductivity (2.2 × 10^6^ S cm^−1^),^266^ good thermal conductivity (28W m^−1^ K^−1^ at ≈37 °C),^267^ is highly stretchable,^258^, ^262^, ^265^ and reconfigurable due its inherent oxide film that considerably decreases its surface tension without significantly impacting its other properties (Figure 7);^268^, ^269^ gallium oxide thin film thus permits liquid metal droplets to wet polymer surfaces and also enables an inconsequential barrier to form for optimal electrical charge transport. Moreover, the wettable nature of gallium also allows it to adhere to the polymer surfaces to form almost any shape for soft and stretchable circuits. Other gallium‐based liquid metal alloys such as ternary alloys (galinstan; 68% gallium, 22% indium, and 10% tin) and eutectic gallium–indium alloys (EGaIn; 75% gallium and 25% indium) have also attracted much interest, as gallium alloys are easier to shape due to their below room temperature melting points, while displaying otherwise similar physical properties to conventional gallium oxide films.^270^

Due to their unique material properties and below room temperature melting point (gallium melts at 30°), gallium alloys have recently been used as conductive fillers in place of rigid filler particles to improve the electrical and mechanical properties of elastomeric polymers.^271^, ^272^, ^273^ In brief, viscoelastic gallium droplets are shaped into the desired electrical circuits by injection of the liquid metal into premade hollow architectural geometries within elastomers. The gallium‐based circuit is subsequently hardened by freezing the composite system—as a whole—below the melting temperature of the liquid metal solution. Gallium‐based alloys can thus readily be used to generate highly complex stretchable circuits by simple injecting them into such elastomeric materials (Figure 7).^265^, ^274^ For instance, injection of EGaln into hollow poly[styrene‐*b*‐(ethylene‐*co*‐butylene)‐*b*‐styrene] fibers have lead to stretchable and electrically conductive circuits that could stretch up to 800% before mechanical failure, without losing their electrical continuity.^265^ These elastomeric circuits could find widespread importance in many exciting applications ranging from flexible electronics, electronic textiles, stretchable wires, and flexible bioelectronics.

Liquid alloys composed of gallium are also ideal candidates for creating conductive circuits within elastomeric substrates, which spontaneously can self‐heal electrical and mechanical defects imposed on them during wear and tear.^260^, ^275^ A recent study accomplished this daring task through a simple system composed of a hollow self‐healing polymer (Reverslink@) into which EGaln was injected. This marvelous system—in the advent of damage—could spontaneously heal its electrical properties and mechanical properties after 10 minutes. In another recent study flexible galinstan‐based electronics was generated through inkjet printing of the liquid metal onto a stretchable PDMS substrate. Notably, the galinstan‐based circuit could spontaneously heal itself after damage, and was engineered in a manner, that enabled it to remain electrically and mechanically stable even after 2000 stretching cycles at strain of 60%.^276^

The incorporation of liquid metals within polymers and then the deposition of the entire system onto a conductive metal trace is another avenue for achieving a self‐healing circuit, as this ingenuity enables liquid metals to readily reconnect distal parts emerged during tear caused by either wear or mechanical failure.^261^ In perspective, owing to its unique self‐healing properties, stretchability and conductivity, we anticipate that the integration of liquid metals into self‐healing elastomers will pave the way for many exciting opportunities for highly flexible bioelectronics in the near future.

Indeed, the aforementioned multifunctional properties of gallium and its alloys have also made them attractive candidates for bioelectronics. For instance, it has been reported that the EGaIn electrodes can be used to both record and stimulate electrical activity in individual neurons.^277^ Another particularly interesting application utilized the electrical and physical properties of Ga‐based liquid metals to achieve an injectable soft 3D electronic circuit that could be delivered to target tissues, such as heart and sciatic nerve.^278^ In detail, this electrode was made from a Ga_67_In_20.5_Sn_12.5_ alloy encased in a biodegradable gelatin hydrogel to achieve a minimally invasive integration between electrode and tissue. Although gelatin is widely recognized as a biocompatible and degradable polymer, it however is not conductive and stretchable. To this end, we anticipate that it is possible to use other types of polymers such as conductive PEDOT‐based polymers to improve electrical and mechanical properties of the system.

Another interesting feature of the liquid metals is their ability to change shape via a variety of mechanisms such as mechanical and electrical stimuli, because this unique trait could be useful for a number of applications such as reconfigurable electronics and bioactuators. Furthermore, recent studies have also shown that the combination of fluidity, deformation reversibility, and conductivity in liquid metals can yield artificial microrobots that can move through blood vessels or intestines to fulfill numerous biomedical purposes.^279^, ^280^, ^281^, ^282^ For instance, the ability to reshape liquid metals by near‐infrared irradiation has recently been utilized to develop an innovative toolbox for controlled drug delivery and optical manipulation of artificial blood vessels.^282^ These studies have opened a new avenue for the construction of intelligent biorobots that could not be obtained through conventional rigid materials.^282^ Nevertheless, the in vitro and in vivo application of flexible microrobots require rigorous testing before they can be applied to combat and monitor various diseases.

## 5 Healthcare Monitors for Empowering the Patient

**Figure 8:**
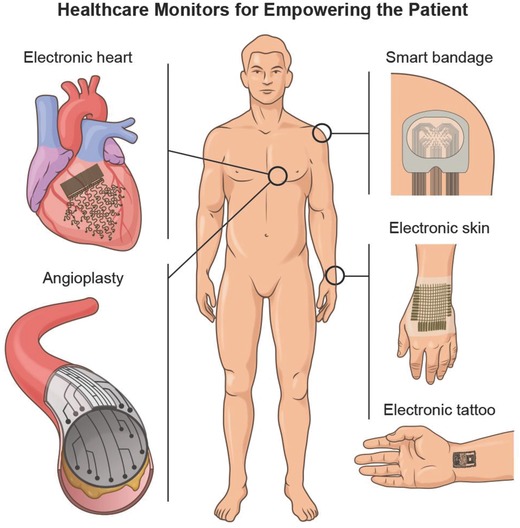
The field of patient empowerment is currently driven by wearable healthcare monitors (e.g., smart bandages, electronic skin devices, tattoo‐based sensors) and implantable monitors (e.g., flexible electrodes for electrocardiography [ECG] and smart stents for angioplasty). Made by Harder&Muller.

Currently, there are a growing number of breakthroughs in bioelectronics driven by the emergence of better and smarter materials that can readily integrate with the dynamic human body. These technological breakthroughs are aiming to empower the patient through technical health aids that enable real‐time monitoring of medical risk factors.^283^ This is accomplished by providing the healthcare consumer with individualized health data for self‐diagnosis and self‐management of their personal health. Such wearable healthcare monitors will also allow doctors to check on patients remotely instead of costly and frequent in‐person visits at the clinic. As a result of their projected importance in the healthcare industry, the market for healthcare monitors is growing quickly, and this year alone it is anticipated that over 19 million of such devices will be sold over the counter and over 100 million devices by the end of 2018.^4^ In spite of the great promise that they hold, the reliability and validity of the data obtained from wearable healthcare monitors are still under intensive investigation. At the moment, there is a lot of ongoing research in the design and development of flexible and biocompatible materials that ultimately can be integrated into the field of bioelectronics to enable more reliable healthcare monitors. Most of the current efforts are directed toward incorporating these materials into devices such as e‐skin,^284^ smart wound bandages,^283^ and tattoo‐based sensors (**Figure** **8**);^285^, ^286^ however, the research and development of materials for invasive and flexible bioelectronics that enable the monitoring of the beating heart and neurological activity in the brain are also slowly gaining momentum.^287^ Here, we will highlight the recent progress in these emerging areas and briefly outline the possible future directions that they may take.

### 5.1 Wearable

#### 5.1.1 Electronic Skin (E‐Skin)

**Figure 9:**
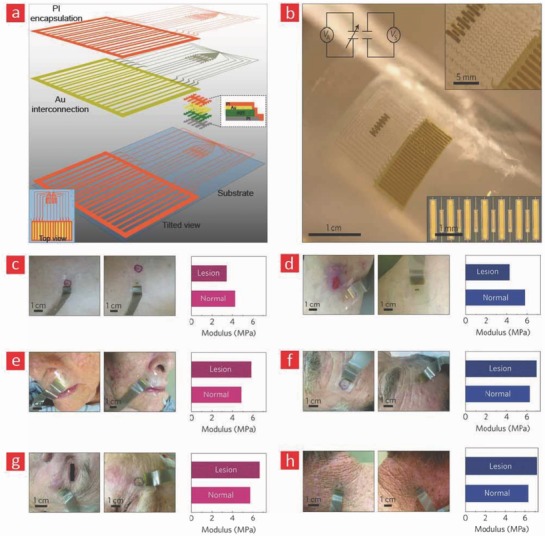
An e‐skin device for monitoring melanoma and skin lesions. a) The device was fabricated through a layer‐by‐layer assembly of Pt/Au electrodes, a piezoelectric component, and a soft and biocompatible PI‐based polymer interfacing the device with the human skin. b) A bright‐field image of the generated e‐skin device. Mechanical mapping of various skin pathologies located c) below the breast, d) on the leg, e) around the nose, f) on the forehead, g) close to the eye, and h) on the neck. Adapted with permission.^289^ Copyright 2015, Macmillan Publishers Ltd.

The human skin can reveal important information about the overall health status of the patient as the mechanical metrics of skin are intimately linked to the circulatory function of the body^288^ as well as various skin‐related diseases such as melanoma, psoriasis, and eczema.^4^, ^289^ Therefore, conformal bioelectronics that enables real‐time monitoring of the mechanical properties of skin can be used to detect potentially life‐threatening and chronic diseases in the home rather than in the clinic.^290^, ^291^ Over the years a wide‐range of these so‐called “e‐skin” devices have been developed for healthcare monitoring purposes.^12^, ^62^, ^284^, ^289^, ^292^, ^293^ In simple terms, e‐skin devices are flexible sensing networks with accurate spatial mapping and detection capabilities that enable unmatched recordings of the mechanical metrics of skin. This utility stems in part from the incorporation of 2D nanoelectronics, organic light‐emitting diodes (OLED), and pressure sensors within biocompatible, conformable, and stretchable plastic‐like materials that can withstand high strain deformations while still maintaining their electrical performance.^290^, ^291^ The nanoelectronics and pressure sensors work in coherence to transform the viscoelastic changes of the skin into electrical signals for OLED processing into pixelated signals.^13^, ^284^, ^293^ In this direction it is essential to use a mode of synthesis that can yield uniform devices, wherein the individual components are matched perfectly within the e‐skin system. Some of the most noteworthy examples of e‐skin devices include an elastomeric pressure sensor based on the incorporation of CNTs within a PDMS substrate,^12^ elastomeric dielectrics integrated within an organic field‐effect transistor for measuring the artery pulse from the wrist,^62^ and a PI‐based system that converts viscoelastic changes of skin into a pixelated and user‐friendly format through an intricate interplay between CNT‐based transistors and OLEDs incorporated within the PI‐substrate.^13^ Building on these results, an e‐skin system was recently developed with the capability of in‐depth characterization of various skin lesions related to invasive melanomas.^289^ This concept could potentially be used for rapid characterization of pathological skin conditions and become a platform for at‐home management of skin‐related diseases (**Figure** **9**).

Recently, more sophisticated circuits have been built to expand the sensing capabilities of e‐skin to encompass changes in temperature,^294^, ^295^ humidity,^169^ and chemical variables.^296^ These additional features enable the patient to detect potentially dangerous foreign bodies from entering the body through the skin and to monitor the effect of various lotions targeted against pathological skin lesions. Moreover, the temperature of the skin is intimately linked to the blood‐flow and therefore presents an important operational parameter that ideally needs to be incorporated into e‐skin devices.^295^ Such multimodal e‐skin devices have so far mostly been based on CNT and graphene‐based nanoelectronics^169^, ^296^ due to the excellent sensitivity of CNTs and graphene toward temperature and humidity changes. To this end, an e‐skin device that is capable of detecting chemical, temperature, and pressure stimuli has recently been developed by sandwiching CNT‐based circuits between PDMS substrates to yield a piezocapacitive system with an ultralow pressure sensitive (0.4 Pa) and fast response time (63 ms).^296^ Notably, this e‐skin system enabled the detection of a range of chemical fluids and could therefore be used as a wearable electronic nose capable of detecting potentially dangerous fluids.

Overall, the abovementioned advancements have led to the development of multifunctional and mechanically robust e‐skin platforms with formidable sensory capabilities for wireless diagnostics.^297^ These advancements highlight the amazing potential of e‐skin technologies and foresee the introduction of e‐skin devices that have the capacity to perceive additional stimuli for various healthcare monitoring.

#### 5.1.2 Smart Wound Bandages

Chronic wounds represent a global healthcare challenge that is expected to grow at a tremendous speed in the coming years as the population ages.^298^ With the current lack of methodology to properly treat the growing number of patients suffering from chronic wounds a cumbersome bottleneck is expected.^298^ This hurdle is intimately linked to today's time‐consuming, costly, and passive wound management scheme, wherein the wound site, is neither monitored nor attended properly. To remedy the current situation, smarter solutions that offer a better insight into the healing process rather than passive wound management are needed. With the recent advancements in biosensors, a new generation of highly sophisticated wound dressings are rapidly emerging to revolutionize the classical wound care concept.^299^ These platforms act as conformal point‐of‐care bandages that consist of sensors capable of detecting important biomarkers of relevance for the wound‐healing process.^300^ They also provide the possibility of remote diagnostics through wireless communication technology and can therefore result in reduced nursing and hospitalization costs.^301^, ^302^

Over the years, a comprehensive list of potential biomarkers for wound healing has been established, which include markers such as pH value, temperature, proteins, inflammatory mediators, cytokines, enzymes, hormones, and nutritional factors.^303^ The most widely used markers are temperature and pH‐value, as they are intimately linked with the extent of inflammation and infection at the wound site.^299^, ^304^ Therefore, a range of temperature and pH sensors have been incorporated into flexible, permeable and biocompatible materials to yield smart wound bandages.^305^, ^306^, ^307^, ^308^, ^309^, ^310^, ^311^ A recent example, is a surgical suture made from silk—a water‐soluble protein—for wound closure and real‐time monitoring of wound‐healing processes. A silicon‐based temperature sensor was incorporated within this silk suture to enable high‐resolution sensing (≈0.2 °C) of temperature changes caused by inflammation at the wound sites.^307^ The system was tested in an animal model and showed promising results that highlighted its potential as a bioresorbable suture capable of monitoring the progress of inflammation within the target site. Building on these results, a multimodal wound bandage was developed with the ability to provide highly accurate temperature readings from the wound site.^308^ The system was multimodal, since it provided readings on both temperature changes and temperature conductivity. As previously mentioned, temperature mapping of the wound site captures the inflammation progress; temperature conductivity, on the other hand, correlates with the moisture content of the wound, which is another important marker that is intimately linked to the state of the wound. In simple terms, this system consists of a PI substrate containing a sensor array that is connected with ultrathin and flexible copper wires. The conformability of the PI‐based device and its capacity to record temperature‐related readings from the wound site were confirmed on human subjects. The reported results were indeed promising and indicated that the developed wound bandage could be readily implemented in a clinical setting.

In addition to monitoring the infection and inflammation status of a wound, other approaches can be explored to promote the healing process by administrating drugs and growth factors to the site. In a recent study by Bagherifard et al.,^312^ thermoresponsive drug‐carriers were incorporated into a hydrogel‐based dressing with a flexible heater for controlled delivery of drugs and growth factors to the wound site. The platform enabled a controlled release of various compounds in response to temperature changes and could therefore potentially be used in smart wound bandages, wherein drugs and growth factors can be released in response to increases in temperature from inflammation and infection.

Bandages with textile‐like materials have also been extensively used for covering wounds, since they are porous, biocompatible, and capable of delivering oxygen and removing exudates from the wound site.^313^, ^314^ They can be fabricated in large scales using well‐known and simple techniques, such as weaving, knitting, and embroidering. Another key advantage of using textiles is that the mechanical properties of individual strands and the entire fabric can be tuned by changing the properties of the fibers or the architecture of the fabric during the manufacturing process.^314^ To this end, a flexible and fiber‐based pH sensor was recently fabricated by loading pH‐sensitive microspheres into alginate‐based microfibers.^23^ The level of acidity at the wound site was measured by taking images of the fibers with a smartphone camera and analyzing the images using an in‐house application. Such hydrogel‐based fibers can also be easily knitted into intricate native‐like architectures and therefore enable the generation of customized bandages for wound‐healing applications. In a another recent study, an advanced wound bandage was developed from conductive threads that were embroidered onto a textile to form interconnected electrodes.^315^ A range of physical and chemical sensors was later incorporated into the bandage to enable high‐fidelity measurements of temperature, glucose, and pH from biological fluids. It was also shown that the developed platform could potentially be used for measuring the physiological conditions at the wound surface through an external device, such as a smartphone or personal computer.

Despite the recent advancements in the development of new smart wound management systems, the available sensors for wearable diagnostic dressings are currently directed toward few physiological markers and thus cannot be specific about the intricate mechanisms occurring in the wound environment. Moreover, the suggested platforms are rarely commercialized and are still in early preclinical stages, choosing the right combination of incorporated sensors and the management of the huge amount of output data is another challenge that needs to be addressed in the future to provide even better wound management systems.

#### 5.1.3 Tattoo‐Based Electrochemical Sensors

Tattoo‐based electrochemical sensors hold significant potential for low‐cost healthcare monitoring applications, thanks to their ability to perform real‐time and noninvasive electrochemical analysis of important biomarkers present in body fluids.^285^, ^292^, ^316^ These wearable sensors, which can be concealed in almost any artistic tattoo pattern, can offer substantial insight into the wearer's health.^317^, ^318^ The majority of the developed tattoo‐based diagnostic devices have so far been based on body sweat due to the wide range of information that it can provide on patient health. This wealth of information include electrolyte imbalance,^319^, ^320^, ^321^, ^322^ bone mineral loss,^323^ presence of heavy metals in the body,^324^ lactate monitoring,^325^ muscular damage,^324^, ^326^ and so forth.

Recently, a range of landmark studies by Bandodkar and Wang has led to the development of a variety of noninvasive tattoo‐based sensors that can effectively monitor several electrolytes and metabolites including sodium,^319^ ammonium,^286^ lactate,^325^ zinc,^324^ glucose,^327^ and 2,4,6‐trinitrotoluene^328^ from human sweat. These advanced tattoo‐based sensors are generated through a conventional screen‐printing technique with smart tattoo inks—onto paper‐based materials made form cellulose—that are reinforced with CNT's to yield mechanically robust devices. Moreover, contrary to their textile and plastic‐based counterparts, these paper‐based sensors developed by Wang and co‐workers demonstrate high mechanical compatibility with skin and thus enable unprecedented skin integration for real‐time chemical and biosensing.^329^

Tattoo‐based wearable sensors have also been developed to monitor electromyography (EMG) signals from the skin surface. The monitoring and subsequent control of such electrical signals have over the years been used for many clinical purposes, such as identifying neuromuscular disorders,^330^ gait disorders,^331^ studying muscle pain,^332^ and also to serve as a control signal for various prosthetic devices.^333^, ^334^ In practice, most EMG skin‐contact electrodes are made from Ag/AgCl gel electrode (pasted with adhesive tapes or straps on skin), however, they are limited in their functionality as they quickly dry out. Other disadvantages are patient discomfort, poor signal transmission at the electrode–skin interface, and the requirement for skin cleaning/preparation.^333^, ^335^, ^336^ Therefore, to overcome these challenges, “tattoo electronics” or “epidermal electronics” was recently applied to create skin‐like EMG sensors with thickness and mechanical properties resembling that of the human skin. One of the first examples of this concept was proposed by Rogers and co‐workers,^284^, ^337^ who packed electrodes, electronics, sensors, power supplies, and communication components into an ultrathin, stretchable, silicone‐based membrane that could attach to skin in a similar vein like a tattoo. This system was tested on almost every part of the human body (forearm, throat, face, forehead, back of the neck, and index finger) and was found to have sufficient quality for measuring EMG signals generated by the contraction of skeletal muscles.^284^, ^333^

Although, such flexible EMG devices are well suited for electrophysiological recordings at various locations on the body, they still face significant challenges related to their stretchability and electrical response time. One noteworthy attempt to remedy this bottleneck was based on using graphene‐based electronic tattoos, wherein graphene was incorporated into a flexible thin (≈463 nm) poly (methyl methacrylate) substrate.^338^ This ultrathin device could readily become attached on human skin via van der Waals interactions and exhibited stable electrical conductivity even when the device was stretched to 50% strain values. For EMG sensing, they placed the device directly on the human forearm without any skin preparation and compared it with commercial Ag/AgCl gel electrodes. The resulting comparison showed that the electronic tattoo–skin interface impedance was almost as low as that of commercial gel electrodes with similar susceptibility to human motion.

Like‐wise, tattoo‐based e‐skin has been developed for controlling human prosthetics by stimulating the muscles in the vicinity of the implant electrically.^339^ To this end, EMG signals generated by muscle contractions of the biceps or triceps have been harnessed by e‐skin tattoos to move artificial limbs. Indeed, we anticipate that such sophisticated systems could spearhead the development of patient‐friendly and less invasive human–machine interfacing with the purpose of controlling the movement of various prosthetic devices.

Further advancements in 3D printing of electronic materials into highly complex circuits are expected to lead to miniaturized and electronically integrated tattoo‐based skin devices with higher mechanical and chemical potency that can cover a broader range of biomedical applications. Moreover, development of tattoo‐based energy harvesting devices^340^ is anticipated to open up new attractive paths toward the fabrication of entirely self‐sufficient tattoo‐based sensors.

#### 5.1.4 Electroencephalography (EEG)

The human brain has always been one of the biggest mysteries in biology and is still in many ways an uncharted territory. Hence, different types of recording systems have been invented to collect information that can elucidate what really lies hidden beneath the skull and deep within the human brain. EEG is one of the gold standards for this endeavor because of its cost‐effectiveness, inexpensiveness, noninvasiveness, and high temporal resolution. In brief, EEG devices record the spontaneous electrical activity along the human scalp to detect neural oscillations from the brain—the so‐called “brain waves”—as abnormal brain wave activity typically coincides with most brain disorders.^341^ Although EEG device has found use in several diagnostic applications including Parkinson's,^342^ schizophrenia,^343^ epilepsy,^344^ and Alzheimer's,^345^ some major challenges still remain unsolved. These challenges include its bulky design, inability to conform to the body over long time periods and the need of a conductive gel at the skin‐electrode interface for efficient electrical coupling.^346^ Other disadvantages are patient discomfort, cumbersome procedures for mounting the electrodes and inability to perform chronic recordings due to drying of the conductive gels. Therefore, new strategies that can reduce the discomfort associated with mounting and wearing the EEG equipment are needed.

One avenue for remedying the current situation is through the development of new and better materials that can yield conformable EEG electrodes capable of adhering onto the human skin without creating any discomfort for the patient. An achievement like this will undoubtedly initiate a more widespread use of EEG devices for many patient empowering applications, including at‐home management of brain‐related disorders, sleep monitoring, and cognitive control. Several noteworthy solutions have already been suggested to initiate this landmark change with the major focus being directed toward the inclusion of soft, conformable, and adhesive electronics into the existing EEG concept.^347^, ^348^, ^349^

**Figure 10:**
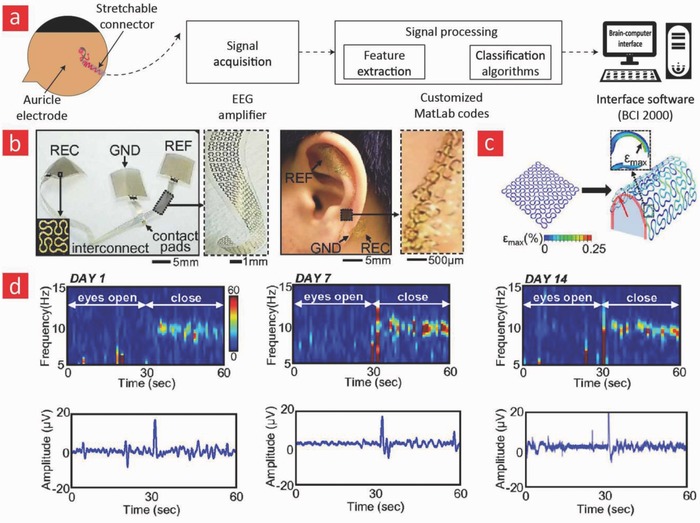
A flexible ear electrode for brain–machine interfacing. a) Schematic illustration of the electrode and the principles underlying the EEG monitoring. b) Images showing the electrode and its mounting on the ear. c) Finitive element method (FEM) analysis of the strain on the device upon mechanical bending (180°). d) EEG alpha wave recordings were fairly stable for up to 14 days after mounting the ear electrode on the user. Adapted with permission.^347^ Copyright 2015, National Academy of Sciences.

To this end, a conductive polymer‐based EEG electrode was recently developed that enables a much better conformal contact with the skin and higher quality recordings compared to gel‐based EEG systems.^348^ The vastly improved electrical coupling with the skin was achieved by the deposition of PEDOT:PSS onto a flexible PI substrate. Interestingly, clinical recordings carried out on human subjects with this new EEG electrode significantly outperformed those retrieved from conventional Au electrodes. In another recent study, a conformable electrode system was developed and ear‐mounted for brain recordings (**Figure** **10**).^347^ The mounting of an EEG device onto a topological complex organ like the ear was overcome by embedding a stretchable and serpentine‐like Au electrode into a flexible and adhesive PI substrate. The serpentine‐like electrode architecture resulted in excellent bendability (>180°) and stretchability (>50%) while maintaining the operational capacity of the EEG device. The intrinsic properties associated with the compliant electrode configuration resulted in high‐fidelity recordings and conformability even during intensive exercise, swimming, showering, and sleeping.

Conformable, durable and user‐friendly EEG electrodes based on flexible, conductive and biocompatible materials are something that the health‐oriented consumer is currently craving, as EEG electrodes are already being used to improve the level of concentration^350^ and cognitive abilities^350^, ^351^ of the user. In the authors' opinion, the commercialization of the abovementioned EEG systems could therefore spark tremendous interest and open up new technology avenues with hitherto unseen consumer empowerment.

### 5.2 Implantable

#### 5.2.1 Electrocorticography (ECoG)

Even though wearable EEG electrodes have provided neuroscientists with a wealth of information over the years, electrodes that are in direct contact with brain neurons enable much higher fidelity recordings in terms of spatial resolution (≈1 cm).^352^ Among the different implantable strategies for monitoring brain activity, ECoG or intracranial electroencephalography (iEEG) has found the strongest foothold, as it is the least invasive option but yet the most accurate. The ECoG concept is based on an electrode that is implanted beneath the skull and in most cases on the surface of the brain. It is used to monitor brain functions in patients with epilepsy^353^ and as a tool to assist surgeons in complicated brain surgeries.^354^ However, the ECoG procedure can be invasive, as the electronic materials used at the moment are inflexible and thus do not conform well to the curvy brain architecture. ECoG devices based on materials that enable them to integrate with the curvilinear surface of the brain could offer a less invasive option for diagnosing, treating, and performing surgery on diseased brains. To address this issue, flexible and soft ECoG implants that can conform to the architectural intricacies of the brain have been developed.^8^, ^9^, ^355^, ^356^, ^357^ One of the most noteworthy attempts to solve this problem was based on a flexible PI‐based electrode with significant spatial (≈500 µm spacing) and temporal resolution (>10 kS s^−1^).^9^ This device was truly a piece of engineering art, as it was capable of incorporating thousands of interconnected silicon‐based sensors to yield exceptional fidelity without losing its flexibility. It was implemented in a feline model and used to record feline brain activity during sleep and electrographic seizures with hitherto superior recordings. In a similar vein, Malliaras and co‐workers developed a flexible electrode array, which was implemented with great success in a rat model. In simple terms, the ECoG device developed by Malliaras and co‐workers,^71^ consisted of a PEDOT:PSS microarrays containing electrodes covering an area of 10 × 10 µm^2^ and with a center‐to‐center distance of 60 µm. This high‐density configuration enabled unprecedented recordings of the electrical activity in rat brains along with good biocompatibility and flexibility. The developed ECoG electrode was also able to record signals that bear resemblance to epileptic spikes in a high‐fidelity manner. Building on these results, an even more dense and flexible electrode that could record action potentials in the brain was developed.^8^ The electrode was coined the “NeuroGrid” and consisted of an ultradense electrode configuration with the area of the individual electrodes being 10 µm × 10 µm and a 30 µm spacing between them. The NeuroGrid was able to perform very high‐resolution recordings of action potentials from the surface of the brain cortex without even penetrating the delicate brain tissue, as was demonstrated both in rat and human subjects. Although, the neurogrid enabled stable brain activity recordings for up to 1 week within a host animal model, its long‐term electrical stability have not been tested yet, and accordingly the feasibility of this approach as a more permanent implantable solution for tracking and treating neurological disorders remains unclear. However, these types of implantable brain recording devices still represent a unique window into the brain and further advancements in this direction could result in breakthrough discoveries in neuroscience and ultimately enable us to better understand and cope with the most vital organ in our body; namely the brain.

#### 5.2.2 Electrocardiography (ECG)

Cardiac‐related diseases remain one of the major health issues in the world and are currently the number one cause of mortality in the developed countries.^358^, ^359^ The ability to record electrical signals generated by the heart is vital for both understanding^360^ and treating^361^, ^362^ such diseases and also for detecting life‐threatening changes in the rhythm of the heart in the home rather than in the clinic.^363^, ^364^ To this end, ECG has emerged as a simple and low‐cost procedure for recording the electrical activity of the heart, as the only requirements are a set of electrodes, which are either placed on the skin or in close proximity of the heart.^365^, ^366^, ^367^ Albeit, electrodes placed on the skin are noninvasive and capable of real‐time monitoring of heart activity, the reliability and validity of the data obtained from such approaches are still under intensive investigation as these electrodes are not directly in contact with the heart tissue and thus unable to precisely record, measure, and stimulate the beating heart and control its electrical rhythm as compared to implantable ECG devices that are put in direct contact with heart tissue.^11^, ^368^ Since most cardiac disorders cause irregular heart beating, the ECG method is a reliable tool for heart diagnosis, as it is pretty straightforward to distinguish between harmonic and nonharmonic electrical recordings from it. ECG is also a feasible method for electrically stimulating the diseased heart in order to mend it again.^369^ These examples include restoring normal heart functions in patients with an abnormal heart rhythm or inefficient heart pumping arising from birth defects, age, or due to myocardical infarction.

Conventional ECG electrodes are typically assembled on rigid substrates with sharp edges and therefore cannot integrate properly with the curvilinear and soft heart tissue to enable the needed electrical coupling between tissue and device for high‐fidelity recordings.^370^ To address these challenges, 1D nanomaterials such as SiNWs,^18^, ^371^ AgNWs,^226^ and CNT^372^ have been incorporated into biocompatible and soft elastomers to develop flexible and less invasive ECG devices. For instance, in a recent study, a silicon‐based nanocircuit was assembled onto a flexible PI substrate (50 µm thickness) to provide robust electrical recordings.^371^ This device was able to conform to a beating embryonic chicken heart while enabling optical imaging and electrical recordings with excellent signal‐to‐noise ratios and high spatial resolution (in the µm range). In a similar vein, a flexible ECG device was recently engineered that consisted of an Au‐based electrode network (total of 288 measurement points) deposited onto an ultrathin (25 µm) flexible PI substrate.^54^ The developed ECG device was implanted in a porcine animal model; thanks to its many electrical contact points (288 measurement outlets), it could record electrical signals with unusually high signal‐to‐noise ratio (≈34 decibels [dB]) and temporal resolutions (<2 ms). It was also readily integrated with the curvilinear and dynamic in vivo heart and displayed a formidable fatigue resistance that enabled the electrodes to maintain their electrical performance even after 10 000 bending cycles.

Although the abovementioned flexible ECG devices are well suited for recording electricity from the curved heart surface, they are incapable of penetrating the many ripples and grooves of its surface, instead they are only able to establish conformal contact with localized regions underneath the heart surface. This current lack of methodology limits the recording and stimulation capabilities of ECG devices, and therefore even more cardiac‐friendly systems are called upon. One of the most obvious attempts to address this challenge is to replace the nonadhesive and biologically stable PI substrate used in most flexible ECG devices with a more conformable, adhesive, and bioresorbable material.^10^ Recent work demonstrates that silk can be used as a highly adhesive carrier material for electronics and transistors, as it is soft, conformable, and displays a degradation profile, which can be fine‐tuned by either controlling the degree of crystalline β‐sheets within the silk film or through enzymatic linkages.^373^, ^374^, ^375^ Hence, silk displays a unique set of properties of high interest for implantable cardiac devices, as it offers exceptional conformability and is capable of transferring and laminating electronic components onto the most rough tissues in the human body.^10^, ^357^, ^376^ On this ground, an electrode network—capable of sensing bioelectricity—has been transferred onto a sacrificial, adhesive and highly conformable silk substrate to engineer an implantable ECG device that fully addresses the abovementioned challenges.^10^ The silk‐based electrode could laminate ECG electrodes onto the most irregular and wrinkled regions of the heart surface, which thus far had been unattainable through conventional strategies. Notably, the laminated electrode array covered the curved surfaces of the heart in a 3D fashion while enabling a robust adhesion onto the surface with stable electrical recordings up to a ≈22% device strain.

An alternative system to the silk‐based material strategies was recently developed to further improve recording/stimulation of the cardiac tissue by using ultrathin elastic membranes that matched the complex heart tissue architecture while simultaneously providing excellent electrical contact with important physiological areas on the heart tissue.^11^ This—next‐generation—ECG device is capable of adhering and enveloping the heart without the need of sutures or tissue glue. Moreover, it is capable of high‐resolution readings of cardiac activity and is able to carry out localized electrical stimulation because of its conformal contact to all points on the heart. Furthermore, a uniform and high‐density distribution of sensing electrodes on the device enabled high‐fidelity electrical recordings from an ex vivo rabbit heart with excellent spatial control. Devices like this could spearhead new innovations in implantable cardiac monitors and stimulators, since they provide formidable flexibility and are capable of maintaining a strong contact with the heart tissue during contraction and relaxation.

In summary, the abovementioned flexible and bioresorbable materials have enabled ECG systems for real‐time monitoring of cardiac activity with the grand goal of treating cardiac‐related disorders, as these materials have improved the devices so they can record electrical signals from previously unattainable electrical points within the ripples and grooves of the beating heart while, at the same time, enabling localized cardiac tissue stimulation to mend potentially life‐threatening chronic heart conditions. However, the stability of such electronic devices within biological fluids remains unclear, since biocorrosion at the electrode/tissue interface and the associated lowering of the device functionalities present a significant bottleneck within the field.

#### 5.2.3 Electronic Stents (ES)

An impaired blood circulatory system is among the major causes of mortality in the world, as it can disconnect vital tissues and organs in the body from receiving nutrients and oxygen.^377^ In most cases, the formation of atherosclerotic plaques in blood vessels precedes these events by blocking the flow of nutrients and oxygen to the inflected body part.^377^, ^378^ To this end, endovascular stent grafting has emerged as the gold standard treatment for patients suffering from diseases that arise from blocked or damaged blood vessels.^379^, ^380^ This procedure works by implanting a small wire‐mesh tube into the inflicted area to enlarge the blocked arteries, support the weakened artery walls, and thus establish normal blood flow to the region that has been disconnected from the circulatory system. These traditional stent implants have, over the years, been made from stainless steel or other flexible and non/corrosive metals.^381^ Although the conventional stents can be used to open blocked arteries, significant challenges remain to improve their biocompatibility and flexibility to enable them to fully meld in with damaged blood vessels. Moreover, an ideal stent device needs to be bioresorbable to avoid the many long‐term complications associated with nondegradable implants. To address these challenges, stents have been developed that are based on degradable polymers or various types of conductive magnesium alloys that altogether are capable of maintaining the structural integrity of blood vessels in a hitherto unprecedented manner and disperse in the body after normality has returned to the damaged tissue.^382^, ^383^, ^384^ Some of these next‐generation stents have also used electronic/magnetic materials and can therefore be programmed to release drugs locally in a time‐dependent manner to prevent inflammation and to get wireless feedback from the progress of clearing blocked vessels.^385^, ^386^ Most of these polymers typically degrade within the body—either enzymatically or through hydrolysis—while the degradation pathway of magnesium is based on corrosion, as magnesium quickly oxidize into corrosive magnesium hydroxide (Mg(OH)_2_) in atmospheric air, which degrades into Mg^2+^ and OH^−^ in the body.^387^

In a recent study, these concepts have been united into a sophisticated bioresorbable stent that is capable of giving real‐time feedback on postsurgery inflammation and blood flow through the targeted vessel.^385^ This electronic stent was composed of a magnesium alloy coated with a degradable polylactic acid (PLA) film in which the conductive magnesium enabled wireless transmission of recorded data, and the PLA was used for controlled drug‐delivery. In this system, the recorded data was first stored in a memory module placed on the stent surface and then wirelessly transmitted to an external storage device with the aid of the antenna‐like magnesium alloy. Furthermore, the mechanical strength and structural integrity of the stent after implantation was maintained for a week. Multifunctional stents like this could spearhead new innovations in endovascular implants, since they provide formidable flexibility, degrade in the body after their job is completed and are able to reduce inflammatory reactions around the stent by providing anti‐inflammatory activity through localized drug delivery.

**Figure 11:**
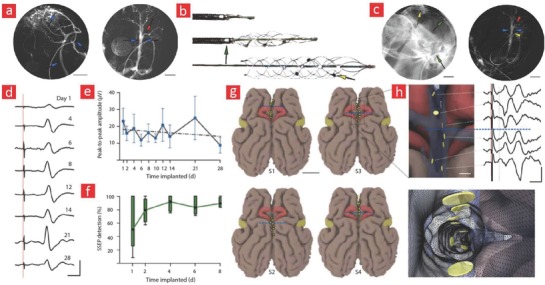
Engineering of an intracranial stent‐electrode (stentrode) array for recording brain activity. a) Preimplant Images showing the presence of intracranial lumens (blue arrow) and cortical veins (red arrow). b) Images showing the self‐expanding property of the stentrode. c) The integration of the stentrode within blood vessels of a sheep brain. The yellow arrows correspond to the electrodes, while the green arrows correspond to the delivery cathers. d,e) The stentrode was able to record high‐fidelity signals following the implantation. The recorded peak‐to‐peak amplitude was fairly stable for up to 28 days. f) Recordings of somatosensory evoked potential (SSEP). g) The position of the implanted stentrode in four different sheep models. h) A 3D representation of an implanted stentrode and its corresponding peak‐to‐peak amplitude recording. Adapted with permission.^391^ Copyright 2016, Macmillan Publishers Ltd.

In addition to the use of stents in treating circulatory related diseases they are also capable of recording and stimulating brain activity from within the narrow capillaries of the brain.^388^, ^389^, ^390^ The keys for successful and noninvasive integration of such stents within the delicate capillaries of the brain are high flexibility and good biocompatibility. To this end, flexible and ultrathin electronic stents represent viable treatment options that can maintain the blood flow into the brain tissue without causing significant damage to the surrounding tissue while at the same time enabling neural recordings.^389^, ^391^ For instance, Oxley and co‐workers^391^ recently developed an electronic stent that could integrate with the ultranarrow capillaries of the brain for both brain activity recordings and neural stimulation (**Figure** **11**). In simple terms, this electronic stent was made from a self‐expanding and commercially available stent coated with biocompatible Pt electrodes for recording purposes and was coined the “stentrode”. The stentrode could readily be integrated within the curved blood vessels of the sheep brain for high‐fidelity recording of somatosensory evoked potentials (SSEPs) in close proximity of the superior sagittal sinus.^391^ This type of electronic stents has also provided a new avenue for deep brain stimulation for up to 190 days with minimal trauma inflicted to the brain.^388^, ^391^ Such implantable systems are indeed safe and can be delivered via conventional catheter angiography and therefore present a minimally invasive option compared with traditional open‐brain surgery.

In summary, the progress made in the field of electronic stents is truly remarkable and will undoubtedly address one of the biggest killers in our times: cardiac‐related diseases. Notably, endovascular stent‐electrode arrays for deep brain stimulation will undoubtedly facilitate breakthroughs in the field of brain–machine interfaces, as they in the authors opinion, will enable seizure prediction in patients with epilepsy and become viable treatment options for those suffering from Parkinson's disease.

## 6 Cybernetic Prosthetics

**Figure 12:**
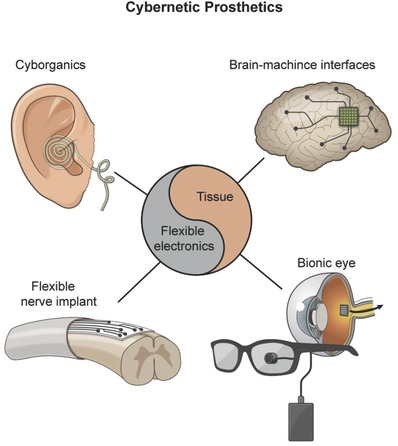
A depiction of some of the most noteworthy cybernetic prosthetics that we anticipate will spearhead the coming cybernetic revolution. Made by Harder&Muller.

Driven by the unification of prosthetics and flexible bioelectronics, the applications of cybernetic prosthetics are numerous and rapidly expanding. Examples include: spinal cord implants that enable the paralysed to move again; brain–machine interfaces that enable the immobilized to mobilize their thoughts to control man‐made limbs; retinal implants that bridge the gap between the brain and the blinded eye to restore vision in the patient; ear implants that make the hearing disabled hear again; and last but not least scientists are following a daring goal of uniting inanimate electronics with living tissues to create cyborganic transplants with unheard of properties (**Figure** **12**). It sounds too good to be true, but yet it is a reality that is currently bringing relief to the lives of disabled people. So far, the primary objective in this regard has been to engineer replacement parts for those disabled since birth through traumatic injuries or from neurological disorders. However, the cybernetic prosthetics could also be used for something entirely different, namely, enhancing human capabilities beyond normality—a topic that raises a host of unanswered ethical questions. Indeed, these are questions that need to be answered before the field of cybernetics can fulfil its true destiny, namely providing the means for mankind to enter the next step in the ladder of human evolution. Among the many key elements that have been used to create these fascinating devices are flexible materials and nanoelectronics. Especially, the union of the two into formidable machines that can integrate seamlessly with the human body has been the driving force behind the aforementioned quantum leaps in cybernetics. The following sections highlight the successful use of these tools by bioengineers to make a cybernetic future implementable in the healthcare system, society, and the private sphere.

### 6.1 Flexible Spinal Cord Implants

Traumatic injuries to the spinal cord significantly deteriorate the quality of life of those affected, and the affected ones typically suffer from chronic paralysis.^392^ In brief, these injuries result in impaired signal conduction between brain and the body and can only be restored through approaches that enable the injured tissue to reconnect with the proper distal target.^393^ One of the key strategies is based on sophisticated nerve implants that consist of microelectrodes that can convey signals from the brain cortex to neurons in a disconnected body part.^394^, ^395^ However, the mechanical mismatch between today's conventional rigid electrodes and the compliant neural tissues significantly limits their long‐term performance and needs to be addressed to fully exploit their clinical potential. In fact, most electronic implants have a young modulus in the GPa range; a stark contrast to the very soft neural tissues of the human body (<1 kPa).^396^

**Figure 13:**
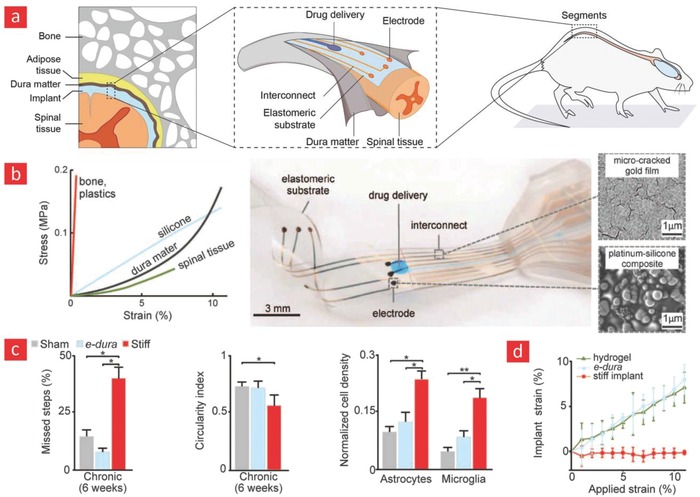
Neural implants with native‐like elasticity. a) Schematics of the electronic dura (e‐Dura) mater implant and how it works in vivo. b) The elastic properties of e‐Dura and various native tissues. c) Postimplantation walking efficiency of rats, the circularity index of the operated spinal cord after 6 weeks, and the density of two cellular markers for foreign body response (astrocytes & microglia) in the spinal‐cord. d) The local longitudinal strain of the e‐Dura increased by much more as a function of applied strain as compared to a conventional rigid implant. This is indicative of a more compliant implant that can cope with the movement of the spinal‐cord region during daily activities. Adapted with permission.^15^ Copyright 2015, AAAS.

The research and development of soft and conformable materials for neural implants is still in its infancy and therefore presents an uncharted territory ripe for fruitful investigations. In a recent cutting‐edge study by a group of scientists from École Polytechnique Fédérale de Lausanne (EPFL), this daunting challenge was addressed by embedding flexible Pt electrodes and interconnects within a soft and biocompatible silicone‐based substrate, which they coined electronic dura matter or simply “e‐Dura” (**Figure** **13**).^15^ The e‐dura implant is composed of a highly elastic silicone substrate measuring 120 µm in thickness, stretchable Au interconnects (35 nm in thickness) and soft electrodes coated with a Pt–silicone composite (300 mm in diameter), which altogether enable the device to mediate electrical stimulation and transfer electrophysiological signals from the spinal cord tissue to a microcomputed tomography (CT) scanner for assessment of whole‐body movements during daily activities. As a new feature, a microfluidic‐based delivery system (100 mm × 50 mm in cross‐section) was incorporated into e‐Dura for controlled delivery of various chemical substances to the injury site. Overall, this system was highly stretchable and exhibited an elastic modulus of ≈1.2 MPa, which is quite similar to the mechanical properties of the native dura mater.^15^ These unique mechanical properties of the e‐Dura allowed it to conform well to the curvilinear surface of brain and spinal cord tissues without damaging them. The e‐Dura implant could sustain numerous stretch cycles without breaking or losing functionality and was shown to restore the motor functions of rats suffering from spinal‐cord‐induced paralysation through a combination of chemical and electrical stimuli. The long‐term functionality and biointegration of the implant is evident, since the new neuroprostheses neither altered the cross‐section of the spinal cord nor triggered unwanted foreign body responses. On the other hand, conventional rigid electrodes lead to significant damage to the spinal cord and failed to completely restore lost motor functions in the operated rats. The e‐Dura implant is the first of its kind and enables the sought‐after long‐term application of spinal cord electrodes. It has huge potential, and it will be interesting to see how it will function in human trial studies. A successful outcome would undoubtedly encourage the millions of people currently suffering from spinal cord injuries and bring a long‐waited relief to their lives. The e‐Dura implant also holds great promise as a potential neuron‐to‐machine interface for real‐time disease monitoring and pain management for patients suffering from neurological disorders.

### 6.2 Noninvasive Brain–Machine Interfaces

**Figure 14:**
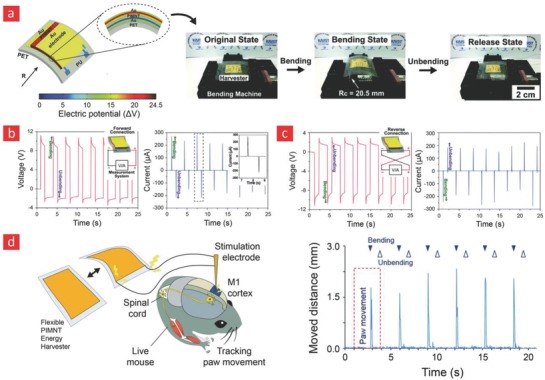
Self‐powered brain–machine interfaces. a) Schematics of the piezoelectric energy harvester and photographs showing the device in its original, bending, and release state. b) The electrical signal measured from the device during bending and unbending in the forward connection and c) reverse connection. d) Depiction of the animal experiment, and data related to the stimulation of paw movement of mice through bending and unbending of the flexible energy harvester. Adapted with permission.^404^ Copyright 2015, the Royal Society of Chemistry.

While spinal cord electrodes aim to restore mobility in paralysed patients by reconnecting the lost motor area with the brain cortex, brain–machine interfaces achieve this by rerouting motor‐related signals to manipulate prosthetic limps.^397^ In general, brain–machine interfaces are sophisticated pieces of equipment that are capable of receiving and delivering feedback related to control and movement of various body parts. Their primary function is, therefore, to establish a communication link between neural activity in the brain and distal body parts. This concept can also be used to control inanimate objects with the mind through a communication link between the brain–machine interface and electronics inserted within the host.^398^ Examples include opening doors, controlling the lighting in a room and moving an item from one place to the other. Brain–machine interfaces have also demonstrated the potential to treat a wide variety of neurological and psychological disorders, such as Parkinson's disease,^399^ Alzheimer's disease,^400^ psychiatric disorders,^401^ and multiple sclerosis.^402^ This is achieved through deep brain stimulation with electrodes that regulate and redirect abnormal neural impulses in the brain.^9^, ^403^, ^404^ In an interesting example from 2015,^404^ a self‐powered brain–machine interface for deep brain stimulation was introduced (**Figure** **14**). The interface was completely self‐sufficient, and it used a flexible piezoelectric component to harvest energy from mechanical motions in the body. It also showed promising results in terms of harvesting sufficient energy to efficiently stimulate the motor cortex inside the brain of mice. Self‐powered deep brain stimulators can address many issues related to conventional battery‐driven implants, because they are 100% self‐sufficient and can therefore circumvent the many invasive interventions that battery‐driven brain–machine interfaces require in terms of repeated battery changes.

However, unfortunately, many of the above‐referenced brain–machine interfaces do not meld well with the human brain due to a mechanical and biological mismatch.^405^ This is possibly a result of the inability of rigid electrodes to conform to the swelling and contraction of the human brain during day‐to‐day activities.^9^ Over the years, several studies have addressed this challenge through flexible and soft electrodes that can integrate easily with compliant brain tissue.^406^, ^407^

**Figure 15:**
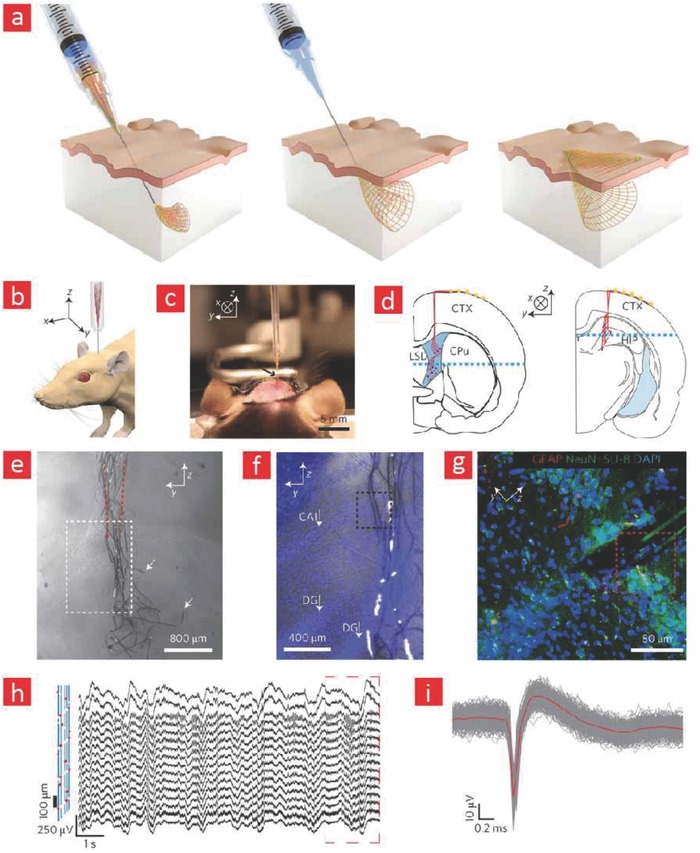
Syringe‐injectable electronics for neural recording. a) Depictions of syringe‐injectable electronics. b) Schematics showing the injection of the electronics into the brain of mice. c) Photographs depicting the injection process into a three‐month‐old mouse brain. d,e) Schematics showing the areas of the mice brain, wherein the mesh electronics was injected. f) Bright‐field microscopy imaging of the brain region into which the mesh electronics was injected five weeks after injection. g) Bright‐field and epi‐fluorescence images corresponding to the region indicated by a white box in (f). h) Fluorescence image corresponding to the region indicated by a blue box in (f). i,j) Electrical recordings from the mouse brain using the injected mesh electronics. Adapted with permission.^409^ Copyright 2015, Macmillan Publishers Ltd.

Although flexible brain–machine interfaces hold great promise as a less invasive treatment than their rigid counterparts, they still rely on invasive and costly surgical procedures. To address this challenge, a method is required that is completely noninvasive to enable a seamless merger between flexible electronics and the brain. To address this grand challenge, a methodology was recently introduced by Lieber and co‐workers,^408^, ^409^ which was based on the delivery of flexible silicon‐based circuits into the brain for monitoring and recording neuronal activity. The process involved a thin syringe needle loaded with freestanding and flexible electronic components that were injected into the target tissue (**Figure** **15**).^409^ Once inside the body, the flexible electronics could reshape into the desired layout and yield high‐resolution recordings of brain activity. The unique structural and mechanical property of the injected mesh electronics also resulted in an unusually facile integration with the brain tissue, which displayed an unmatched portfolio of formidable properties once inside the brain, as it was more conformable and smaller than any other electrodes that have been implanted into the brain. This cutting‐edge technology will unequivocally lead to a momentous advance—not only as a platform for more sophisticated brain–machine interfaces—but also as a new technology with the capacity to reshape the entire field of biomedical engineering.

### 6.3 Retinal Implants

Age‐related and disease‐related degeneration of the human eye affects millions of people worldwide and is expected to grow rapidly in the coming years due to the ageing global population.^410^, ^411^ Retinal prosthesis provides an opportunity to restore the vision in those who have lost sight through such degenerative diseases.^412^, ^413^ In brief, a retinal prosthesis replaces the function of the damaged eye by exciting nerve impulses from healthy neurons in the eye; this is accomplished with the aid of microelectrode arrays on the prosthesis. The excited nerve impulses are subsequently transmitted to a chip in the virtual cortex of the brain to create artificial visions of the surrounding world.^414^ In order to prevent physical damage to the retina at the implant–tissue interface, it is desirable to have implants made from materials with properties such as bendability, foldability, and biocompatibility.^415^, ^416^ Currently, most retinal electrodes are made from silicon‐based microelectronics^416^, ^417^ with relatively good biocompatibility. However, the silicon‐based electrodes can be mechanically rigid and bulky,^416^ and they are difficult to mold into minimally invasive and ultrathin configurations. Therefore, these substrate types could in the long run lead to severe tissue damage due to a mechanical mismatch between the rigid implant and the surrounding retinal tissue. PI‐based electronics have emerged as promising flexible materials for retinal prosthesis, as they both can accommodate for the movement of the eye during day‐to‐day routines and are compatible with native tissues.^418^, ^419^ PI's can accomplish this daring task, since they are biocompatible and moldable into the ultrathin and delicate electronic devices required of retinal prosthetics. They also recover to their original shape even after rolling or folding in stark contrast to silicone‐based electronics.^420^, ^421^ Furthermore, other type of polymers such as parylene C and PET have been proposed for retinal prosthetic, however, they have higher CTE and lower *T*_g_ compared to PI, as described in Section 2.2 (PI) and Section 2.3 (PET and parylene C).^422^, ^423^ Therefore, they have much more difficulties with withstanding the high processing temperatures during the manufacture of metal‐based electrode arrays. Importantly, a high CTE can also facilitate strain accumulation within the polymeric film and thereby increase the risk of crack formation between the electrode layer and substrate at elevated temperatures.

One noteworthy example of PI‐based retinal devices is the Argus II, which consists of 60 Pt microelectrodes embedded on a flexible PI‐substrate.^16^, ^424^, ^425^ The Argus II was the first prosthetic retinal device to receive regulatory approval in both USA and Europe^16^ and is currently an available and commercial option for treating people with impaired eye vision.^16^, ^426^ At the time of this writing, more than 100 retinal devices have been implanted worldwide;^16^ nonetheless, some challenges are still being investigated or remain unsolved at present, and thus further research is required in this direction. For instance, with the Argus II system, it is difficult for patients to perform complex visual tasks, such as face recognition, orientation in unknown environments or text reading.^425^ Therefore, we anticipate that future research will focus on the design and fabrication of material platforms that can incorporate even more electrode units and thus increase the number of excited retinal neurons to obtain the needed complex signaling for complex visualization.

### 6.4 Cyborg Organic Constructs (Cyborganics)

Whereas the aforementioned applications of flexible electronics were primarily directed toward establishing conformal electrical contacts with tissues in vivo to restore and/or enhance the functions of the human body, even more impressive cybernetic systems are currently under development to create cyborg organic constructs (cyborganics). In its essence, “cyborganics” represents an emerging concept at the crossroads of tissue engineering, materials science, electronics, and chemistry with the daring aim to create a new class of hybrid organs and hydrogel carriers made from a combination of inanimate and biological matter.^17^, ^18^, ^427^ Cyborganics can be divided into two different classes:^427^ i) permanent hybrid organs consisting of stable matter and living tissue and ii) hydrogel carriers embedded with biological matter and degradable nanomaterials with the grand goal of regenerating dysfunctional tissues. The concept was recently exploited by Liebers and co‐workers at MIT in a landmark study, wherein stem cells were matured into functional tissues within a web of silicon nanowires.^17^ These remarkable hybrid constructs were capable of real‐time monitoring of markers related to tissue growth and functions. To probe the physiological environment of the living tissue, silicon nanowire FETs, which are capable of recording physiological signals, were embedded within the hybrid constructs. The sensor network was a truly outstanding detector of important physiological events once the encapsulated stem cells had turned into cardiac tissue and premature blood vessels, as it enabled electrical recordings from the beating cardiac tissue with incredible resolution (≈ms) and PH‐recordings from within the vessels.

**Figure 16:**
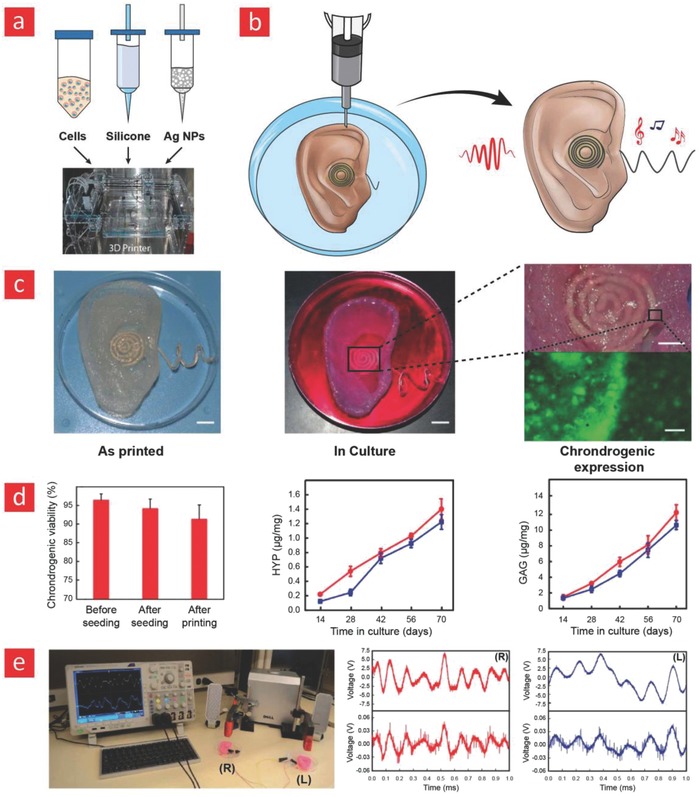
A cyborg ear for enhanced auditory sensing. a,b) Schematics showing the 3D printing of the artificial ear. c) Photographs of the printed ear before and after culturing. d) Chrondogenic cell viability and secretion of important chrondogenic markers. e) Audio signals were transmitted through the right ear (R) and received through the left ear (L) with good fidelity. Adapted with permission^428^ Copyright 2013, American Chemical Society.

The concept of “cyborganics” was also recently applied to create cochlea‐shaped electrodes consisting of cartilage tissue interweaved with inductive coil antennas (**Figure** **16**).^428^ This hybrid implant had similar properties to a native ear and was capable of auditory sensing in the radio frequency range. In brief, an ear‐like construct was generated via additive manufacturing of cartilage cells and Ag nanoparticles embedded within an alginate hydrogel matrix. The three components were merged into the syringe‐extrusion of a 3D printer, which deposited the mixture into a bioelectronic hybrid ear. Over time, the alginate matrix was degraded and the cartilage cells retained the ear‐shaped morphology that was originally 3D printed. Indeed, this concept of additive manufacturing of living cells with electronic materials is leading a highly innovative direction in the field of cybernetics. Still, the generation of such hybrid constructs is still a big challenge in the field. In the authors' opinion, this concept deserves much future attention, as it could enable the generation of a wide range of replacement parts with performances beyond what human organs typically are capable of delivering.

## 7 Outlook

### 7.1 Flexible Materials and Electronics

Flexible and conductive polymers will unequivocally reshape the field of bioelectronics and herald a new age in modern electronics. However, there are many challenges that need to be addressed, since the thermal stability, solvent resistance, CTE coefficients, and conductivity of polymer materials still differ a lot from their inorganic counterparts.^26^ Perhaps the most promising approach to remedy the current situation is through the incorporation of multifunctional nanomaterials into polymers. Polymer materials that are stronger than steel, optically transparent, flexible, and highly conductive have been developed through the unification of nanomaterials and polymers.^225^, ^427^, ^429^, ^430^, ^431^

In one paramount study, carbon nanotubes functionalized with Ag nanoparticles were incorporated into a matrix of polyvinylidenefluoride and solvent cast into an ultrathin sheet (<140 µm). The hybrid film displayed unusual electromechanical properties with a conductivity at 5710 and 20 S cm^−1^ at 0% and 140% strains, respectively.^225^ Building on these results, a microscale paper‐cutting approach, based on a top‐down plasma etching technique, was developed to engineer even higher plasticity into carbon‐based nanocomposite polymers.^432^ Through this approach, the ultimate strain of the nanocomposites was increased from 4% to 370% while preserving their high electrical conductance. This unique property stems from stress delocalization mediated by the notches introduced into the film through the paper‐cutting process. In other studies mineral‐based platelets such as laponite, sumecton, and montmorillonite have been successfully incorporated into various polymer matrixes to yield films with incredible barrier properties and CTE coefficients similar to that of glass.^430^

Moreover, properties such as high‐field‐effect carrier mobility and long‐term stability under mechanical, electrical, and environmental stress have made inorganic semiconductors and nanomaterials suitable for various flexible electronic applications.^273^, ^433^ In recent years, they have also been integrated into a great variety of organic polymer matrices to yield multifunctional flexible devices with improved electrophysiological sensing capabilities.^284^, ^339^, ^434^ Notably, these properties and functionalities of inorganic components are greatly affected by their crystal structure, phase, size, shape, and their chemical attributes.^217^ We envision that the integration of inorganic components into biointegrated systems represent an interesting area ripe for investigations, and could therefore enable significant breakthroughs in the field.

Despite these encouraging results, multifunctional polymers with all of the abovementioned properties are yet to hit the consumer market for flexible electronics. However, the era of nanoreinforced polymers is still in its infancy; we therefore anticipate that further research in this direction will yield polymer composites with even more extraordinary properties than those previously reported.

### 7.2 Healthcare Monitors for Empowering the Patient

The unsystematic healthcare monitoring of the populace is one of the primary driving forces behind many chronic diseases. Examples include the stealthy plaque buildup in blood vessels caused by unhealthy eating habits, the well‐documented correlation between diabetics 2 and high blood glucose levels and the intimate link between a number of debilitating neurodegenerative diseases and unnoticed cases of neuroinflammation. A number of smart technical health‐aids have already been developed with the ability to gather such vital health data from the patients from home without the need of costly and time‐consuming in‐clinic visits. Indeed, Rogers and co‐workers are currently working hard to spearhead some of these technologies into the consumer market through a newly funded company, namely MC10. MC10 specializes in wearable physiological monitors that are made from materials that enable them to effortlessly adhere and integrate onto the skin; they recently launched the “BioStamp,” which is a conformable e‐skin device that can link physiological data recorded from the body to portable devices for efficient readout and analysis of those data. In a similar vein, Google is currently working on a new wrist‐worn device that will enable ECG, skin temperature, and pulse monitoring. In addition to recordings associated with circulatory health and skin wellness it would be interesting to expand the operational capacity of the commercially available healthcare monitors to also include EEG measurements from the brain, similar to the study from Rogers and co‐workers that we reviewed in Section 5.1.4,^347^ wherein a conformable electrode was plugged into the ear for high‐fidelity EEG recordings. We note that such devices, if completed, would constitute complete real‐time healthcare monitors of individualized health data, since they enable heart activity mapping, brain activity recording, and real‐time monitoring of skin‐related diseases.

Stretchable and elastomeric bioelectronics has also been gradually realized in other more specialized sensorial and therapeutic applications. For instance in recent study by Mannoor et al. a flexible material interfaces that can be used to monitor bacterial contaminants on tooth enamel was developed.^435^ The core of this flexible biosensor was an intricate Au‐graphene conduit that was deposited onto a silk‐based substrate through shadow mask‐assisted electron beam evaporation, and subsequently functionalized with antimicrobial peptides. While the printed graphene‐based conduits form the primary “circuit” of the nanosensor, the peptides are aimed to detect appropriate pathogens. In the event of a bacterial attachment to these peptides, the resistance of the graphene coil changes—thanks to the relatively charged cellular membrane of the bacteria—and elicits an inductive effect, which can be detected via a wireless device that utilizes an impedance analyzer. This platform opens up a new paradigm for monitoring pathogens and could find applications in food industry, hospitals, implantable personal healthcare devices, and many more.

In another recent study, a multifunctional and transparent endoscope capable of temperature/pH sensing, drug delivery, and performing real‐time optical imaging was developed.^436^ This transparent electronic system consisted of 1) a graphene and Au‐based sensor that can detect pH and temperature fluctuations in tissues, 2) light sensitive dyes that can be used for imaging purposes, and 3) drug‐loaded nanoparticles with therapeutic potential. The system was implemented in a mouse model and displayed remarkable potential for diagnosing and treating colon cancer in patients.

In summary, there is currently a push in the field of bioelectronics driven by the emergence of a new class of flexible electronic materials, and in the authors' opinion, it seems like its worldwide applicability is closer to becoming a reality than ever before. As this trend continues, we envision a truly remarkable healthcare revolution in the form of personalized health monitoring devices with impeccable accuracy.

### 7.3 Cybernetic Prosthetics

Extensive work on animals has paved the way for implementing a wide range of the aforementioned cybernetic extensions in human subjects. However, a large amount of work is still needed in this direction to develop even better material interfaces to reach the grand goal of realizing human cyborgs with supernatural abilities and powers. Especially, the engineering of electrode materials that can bridge between the brain and human consciousness as well as more sophisticated cyborganics are called upon to bring cyborgs out from the realm of science fiction and into real‐world applications.

Among the most noteworthy applications of brain–machine interfaces is to treat and possibly cure neurological and physiological disorders. Malfunctions in the neural circuitry of the brain and neurotransmitter imbalance are key factors behind these kinds of brain disorders. One avenue for restoring order in the disordered brain is, as briefly mentioned in Section 5.2, to use brain–machine interfaces for electrically stimulating the brain into a balanced state again. Building on this concept, the field of optogenetics has emerged to enable even better control and stimulation of the brain through light‐activated release of neuroactive reagents from neurons that have been genetically altered to become sensitive to light.^437^, ^438^, ^439^ Despite being a relatively young field of research, optogenetics have already facilitated many groundbreaking discoveries. Most noteworthy is its application in systems that can be used to control the motion behavior of transgenic insects.^440^, ^441^ In brief, these systems consist of optically transparent insects with modified brain neurons that can release important behavioral molecules from the brain in response to light‐activation. However, this form of brain stimulation is less straightforward in larger animals, and therefore other approaches are called upon to open the light‐activated gateway into the brain of more complicated organisms. To address this challenge, optogenetic systems based on flexible, light sensitive, biocompatible, soft, and mechanically compliant electrode materials have been developed.^442^, ^443^, ^444^ In a recent example, flexible and wirelessly controllable LED devices were implanted beneath the skull of mice to stimulate dopaminergic neurons to release dopamine.^442^ This study demonstrated that the movement of mice could be easily controlled and re‐directed with the aid of light and subsequent release of dopamine. Overall, these results clearly show that it is feasible to use such microelectrodes for controlling human behavior, curing various physiological disorders and facilitating cognition enhancement in a hitherto unprecedented manner. However, in the authors' opinion, we need to approach these technologies with caution and carefully evaluate the risk and opportunities associated with their use in human subjects, as they can be misused in an unethical manner.

So far, the prime‐usage of cyborganics has been in various diagnostic and proof‐of‐concept applications. However, the union of electronic materials and living tissues can pave the path for impressive devices that can replace dysfunctional organs and enhance bodily functions beyond normality. We anticipate that 3D printing of living cells along with 2D nanomaterials such as graphene or CNTs can yield even stronger tissue constructs that are part animate and part inanimate. Moreover, the deposition of electronics and cells into architectures similar to those found in native tissues through various microprinting techniques is a feature that definitely will spark unprecedented progress in the field of cybernetics.^427^, ^445^, ^446^ Along these thoughts, Shin and co‐workers recently managed to 3D print a mixture of cardiac cells and CNT‐based circuits into flexible electronics with cyborganic‐like features.^445^ Specifically, it was shown that cardiac cells embedded within these hybrid constructs were viable and able to express important cardiac markers. This technology can in the future be expanded to yield cyborg cardiac‐like patches or maybe even spearhead the next‐generation of pacemakers. The prospect of 3D printed hybrid constructs consisting of neurons and flexible microelectrodes could also result in less invasive and more advanced brain–machine interfaces, wherein the fine line between machine and brain is abolished due to the presence of both native‐like brain matter and electronics.

Given the fact that muscle tissue consists of highly aligned and organized muscle bundles, the combination of 3D printed carbon‐made fibers and muscle cells into anisotropic tissue‐like architectures can lead to alternative treatments for those suffering from scleroses and muscular dystrophy. These cyborg muscles are also anticipated to give the wearer formidable strength, similar to the supernatural abilities of the cyborgs, that have been presented to us through science fiction movies.

Ultimately, the engineering of flexible electronics that can blend right in with the human body has driven the field of cybernetics to reach the capacity to deliver truly outstanding human–machine interfaces, that in the authors opinion will yield more advanced and versatile beings in the foreseeable future.

## 8 Conclusion

The widespread usage of sophisticated prosthetics such as cochlea, retina, brain–machine, and neural implants along with the projected importance of technical health aids in our daily‐life‐activities clearly indicates that we are slowly entering a cybernetic era. These advances are a direct consequence of flexible and electronic materials that can meld seamlessly with the soft and curved tissues of the body. The development of brain–machine interfaces that enable thought‐controlled movement of objects has especially stirred a lot of frenzy in the field and we anticipate that these cybernetic extensions could potentially expand human cognition to a new level.

Another emerging topic (i.e., cyborganics) can change the way synthetic material parts and tissue transplants are viewed and ultimately herald a new era in tissue engineering, wherein more durable, better, and stronger tissues are custom‐made in the laboratory to hack the biology of humans beyond our current imagination. In the authors' opinion, further advances in this direction are intimately linked with 3D printing of hybrid neural circuits and nanoreinforced designer materials that can impose exceptional abilities on the user. The advantages that the 3D printing technology bring to the field of cybernetics are first localized printing of cells, materials, and nanoelectronics onto flexible substrates, and second the ability to custom‐make cyborg organs with native‐like tissue architectures. Other areas of research ripe for investigations are cybernetic devices that can harvest energy from the moving body without the need of any interventions or revision surgeries to replace old batteries.

In summary, the ongoing progression of research in the field of cybernetics has been made possible from the beautiful trinity between biology, materials science, flexible electronics, and tissue engineering. However, we should be wary of the fact that the implementation of some of the suggested cybernetic inventions need to be carefully and critically evaluated to make sure they follow existing ethical guidelines.

## Conflict of Interest

The authors declare no conflict of interest.
